# Lipidomic Analysis of Cervicovaginal Fluid for Elucidating Prognostic Biomarkers and Relevant Phospholipid and Sphingolipid Pathways in Preterm Birth

**DOI:** 10.3390/metabo13020177

**Published:** 2023-01-25

**Authors:** Se Hee Hong, Ji-Youn Lee, Sumin Seo, Bohyun Shin, Cho Hee Jeong, Eunbin Bae, Jiyu Kim, Donghee Lee, Byungchan An, Minki Shim, Jung Hoon Shin, Dong-Kyu Lee, Young Ju Kim, Sang Beom Han

**Affiliations:** 1 College of Pharmacy, Chung-Ang University, Seoul 06974, Republic of Korea; 2 Department of Obstetrics and Gynecology, Ewha Medical Research Institute, College of Medicine, Ewha Womans University, Seoul 07984, Republic of Korea

**Keywords:** cervicovaginal fluid, preterm birth, lipidomics, vaginal microbiome, sphingomyelin, biomarker

## Abstract

Cervicovaginal fluid (CVF) is an excellent specimen for monitoring preterm birth (PTB) as it characterizes cervical metabolites, the vaginal environment, and specific host immune responses. However, extensive lipid analysis of CVF to explain PTB has not been studied. In this study, we performed a systematic analysis combining high-throughput lipid analysis and omics to discover the unique metabolic properties of the cervix. Liquid chromatography-high resolution mass spectrometry successfully detected a total of 190 lipids in the CVF of 30 PTB and 30 term birth (TB) pregnant women. The whole lipidomics dataset analyzed by combining multivariate and univariate statistical analysis revealed 35 lipid biomarkers, including phospholipids and sphingolipids. Remarkably, sphingomyelin, which plays a physiologically essential role in sphingolipids, was significantly downregulated in PTB. Metabolic pathway study provides a close relationship between vaginal microbial organization and cell membrane formation, further supporting the robustness of our findings. Sphingolipids and phospholipids, which were determined to be important lipids for predicting PTB in our study, showed a high value of receiver operating characteristic (ROC) curve >0.7, indicating that a lipid diagnostic test and understanding the mechanism of lipids is highly related to the vaginal microbiome. Therefore, our result has high potential as a predictor of PTB.

## 1. Introduction

Preterm birth (PTB), which is defined as gestational age less than 37 weeks, is a serious health problem for neonatal and maternal health with the risk of mortality and morbidity [1]. With the increase in the rate of preterm birth due to the use of assisted reproductive technology or certain abnormalities of the reproductive organs, the need for predictive techniques for premature birth has emerged [2]. Early detection of premature birth risk in women can provide ample opportunity to adopt appropriate intervention strategies to improve maternal and neonatal outcomes [3,4]. Unfortunately, current methods for estimating the risk of preterm birth have low positive predictive values (21% cervical length, 17% cervical fetal fibronectin) and low specificity (52% cervical length) [4]. For this reason, understanding the physiological and pathological mechanisms behind preterm birth is critical to better anticipate and intervene in preterm delivery.

Low-molecular-weight biomolecules in biofluid are one of the most effective potential biomarkers that directly represent the homeostatic change, and thereby, can diagnose gynecological diseases. Many studies elucidated lipid or metabolite indicators of maternal diagnosis in biofluids such as amniotic fluid, maternal urine, and plasma [5]. Previous studies with those samples discovered the metabolite biomarkers for gestational diabetes mellitus (GDM), premature rupture of membranes (PROM), and preterm delivery (PTD) [6]. Urine and vaginal fluid, having simple and non-invasive collection methods despite the rich information inside these samples, have also been used in many diagnoses [7]. The other options include the use of non-traditional matrices (e.g., breast milk, meconium, cord blood, amniotic fluid, or saliva), which may be of interest for assessing prenatal exposures or for specific purposes [8,9,10].

Among them, cervicovaginal fluid is excellent for maternal monitoring because cervical metabolites and metabolite profiles are related to the characteristics of the vaginal ecosystems, specific host immune response, and PTB [11]. Investigation of the metabolites in CVF can provide insight into cellular metabolism associated with the host and the pre-existing microbiota associated with premature cervical remodeling [12]. In addition to the effects of short cervix in the second trimester, Burris et al., investigated the association between cervical microRNA expression and spontaneous preterm birth [13]. Another study performed metabolic profiling of CVF using nuclear magnetic resonance (NMR) spectroscopy and identified metabolic markers to predict PTB [14]. In addition, studies on preterm birth and lipids, studies on sphingolipids and immune relationships, and evaluation of cytokines and prostaglandins in CVF were also conducted [15,16,17]. Despite these studies, extensive lipid biomarkers that account for the diagnosis of preterm birth have not been studied.

Several gynecological diseases have been shown to have a close relationship with abnormal lipid metabolism in body or microbiomes. It is essential that the maternal lipid metabolism is properly controlled for healthy pregnancy and fetal growth [15,18,19]. In previous research [15], as gestational age at delivery decreased (premature birth before 34 weeks), overweight women’s early pregnancy concentrations of total cholesterol (TC) and low-density lipid cholesterol (LDL-C) increased linearly. In addition, with rising TC, triacylglycerols (TG), and LDL-C levels and falling high-density lipid cholesterol (HDL-C) levels, a trend toward an increase in the incidence of unfavorable pregnancy outcomes was seen [18].

Lipids play an important role in a variety of biological processes, from forming the basic units of membrane structure and energy storage, to powerful signaling molecules [20]. For example, polymerized glycerides, such as triglycerides, are stored in fat cells and released via hormonal signals for energy production [21]. Phospholipids, on the other hand, have a polar head covalently attached to a non-polar hydrocarbon tail, making them a key structural component of lipid membranes of vagina or vaginal flora [22]. Sphingolipids are involved in the regulation of inflammation and immune responses during pregnancy [23,24].

In this study, we performed untargeted lipidomics using liquid chromatography-quadrupole-time-of-flight (LC-Q-TOF) to investigate lipid changes between CVFs collected from pregnant women with preterm birth and term birth conditions. Liquid chromatography was the most used method for lipid profiling for screening a wide range of lipid types [25,26,27]. Predominant lipid classes including phospholipids, glycerolipids, and sphingolipids were detected and quantified based on high-throughput lipid profiling. A total of 190 lipids were successfully detected, and they were utilized to understand the role of lipids in pregnancy and their influence on the preterm birth.

## 2. Materials and Methods

### 2.1. Chemicals and Reagents

LC-MS grade acetonitrile, methanol, water, isopropyl alcohol, and HPLC grade methanol, and chloroform were purchased from Thermo Fisher Scientific (Fairlawn, NJ, USA). LC/MS grade eluent buffers, ammonium acetate, and acetic acid were purchased from Sigma-Aldrich (St. Louis, MO, USA). Diacylglycerol (DG, 10:0/10:0), triacylglycerol (TG, 17:0/17:0/17:0), phosphatidylcholine (PC, 10:0/10:0), phosphatidylethanolamine (PE, 10:0/10:0), sphingomyelin (SM, 18:1d/17:0), and ceramide (CER, C17) for internal standards were acquired from Avanti Polar Lipids (Alabaster, AL, USA). Diacylglycerol (DG, 16:0/16:0), phosphatidylcholine (PC, 16:0/16:0), phosphatidylethanolamine (PE, 16:0/16:0), lyso-phosphatidylcholine (lyso-PC, 18:0), lyso-phosphatidylethanolamine (lyso-PE 16:0), triacylglycerol (TG, 17:0/17:0/17:0), sphingomyelin (SM, 18:1d/17:0), and ceramide (CER, C17) as standards for retention time were obtained from Avanti Polar Lipids (Alabaster, AL, USA).

### 2.2. Study Subjects

In a nested case-control study, 60 pregnant women with PTB (gestational age <37 weeks, n = 30) and TB (gestational age ≥37 weeks, n = 30) were enrolled for the CVF analysis during the period 2019–2021, at Ewha Womans University Mokdong Hospital and Hanyang University Hospital, Republic of Korea (Ethical Research Committee approval no. EUMC 2018-07-007 and 2018-09-009). CVF samples were collected by vaginal swab from 20 patients in the second trimester and 40 patients in the third trimester. Coincidentally, equal proportions of pregnant women sampled for each trimester had preterm and full-term deliveries. The clinical characteristics of the subjects are summarized in Table S1.

### 2.3. CVF Samples Extraction

CVF samples were stored at −70 °C until analysis. Before the analysis, the frozen CVF samples were thawed at room temperature and vortexed. Internal standards solution of diacylglycerol (DG, 10:0/10:0), triacylglycerol (TG, 17:0/17:0/17:0), phosphatidylcholine (PC, 10:0/10:0), phosphatidylethanolamine (PE, 10:0/10:0), sphingomyelin (SM, 18:1d/17:0), and ceramide (CER, C17) were diluted with isopropyl alcohol (5 mg/L) and spiked in 30 µL of each CVF sample. The mixed samples were extracted by modified Folch’s method, as described in a previous study [28,29,30,31]. 300 µL of cold MeOH/CHCl_3_ (1:2, *v*/*v*) was added to the CVF sample, and the mixture was sonicated (Daihan Scientific, Seoul, Republic of Korea) for 30 min. After sonication, 200 µL of water was added and the extracted CVF sample was centrifuged at 1350× *g* (CF-10, Daihan Scientific, Seoul, Republic of Korea) for 15 min. The whole process was repeated twice and after the final centrifugation, the lower layer was transferred into a 1.5 mL Eppendorf tube, evaporated under the gentle stream of nitrogen gas, and reconstituted with 100 µL of isopropyl alcohol. The final solution was filtered with a 0.2 µm syringe filter and injected into the LC-MS instrument. The QC samples consisted of equal volumes from all CVF samples and analyzed once for every five samples in the batch.

### 2.4. LC-MS Condition

Lipid profiling of CVF was conducted using Agilent 1290 Infinity system equipped with Agilent 6546 quadrupole-time-of-flight mass spectrometry from Agilent Technologies (Santa Clara, CA, USA). The ACQUITY UPLC BEH C18 Column (2.1 × 100 mm, 1.7 μm, Waters, Milford, MA, USA) assisted lipid separation under a binary gradient elution with a flow rate of 0.15 mL/min at 40 °C. Mobile phase A was acetonitrile/water (2:8, *v*/*v*) and mobile phase B was isopropanol/acetonitrile (6:4, *v*/*v*), both including 10 mM ammonium acetate and 0.1% acetic acid. The solvent gradient condition was programmed as follows: 0.0–3.0 min, 40% B; 3.0–10.0 min 85% B; 10.0–23.0 min 90% B; 23.0–28.0 min 100% B; 28.0–38.0 min 100% B; 38.0–40.0 min 40% B. The injection volume was 5 µL. Separated lipids were detected by electrospray ionization and Q-TOF detector with parameters as follows: sheath gas temperature 350 °C; sheath gas flow, 11 L/min; nebulizer, 17 psi, gas temperature 200 °C; dry gas flow, 8 L/min; capillary voltage, 3500 V; nozzle voltage, 1000 V; fragmentor, 175 V. After obtaining chromatograms using untargeted LC-MS analysis with scan mode, targeted MS/MS with the same parameters confirmed a spectral match with the fragment pattern in database.

### 2.5. Lipidomics Data Processing

Data analysis was performed using Agilent Mass Hunter Qualitative Analysis 10.0. and Agilent Mass Hunter Quantitative analysis (Q-TOF) 10.1. The lipids ID process was carried out as described in previous studies [31,32] based on the Lipid Maps “http://www.lipidmaps.org (accessed on 1 December 2022)” and retention time. Lipid Maps was used for the finding the exact mass. Three-dimensional data (including RT, *m*/*z*, and peak area of all lipid features) were extracted to identify lipid metabolites. This method was applied to the clinical samples. After obtaining chromatograms from 60 CVF samples by untargeted LC-MS analysis with scan mode, targeted MS/MS with the same parameters confirmed fragment pattern of each lipid.

### 2.6. Statistical Analysis

Data were imported into SIMCA version 17.0.2 (Satorius AG, Goettingen, Germany) and multivariate statistical analysis including principal component analysis (PCA) and partial least squares discriminant analysis (PLS-DA) were performed. The unit variance scaling was applied to normalize the intensities of lipid features prior to analytical model fitting. The quality of PLS-DA model was evaluated by the values of explanatory parameter R^2^ and predictive parameter Q^2^. To avoid overfitting of analytical mode, thousand permutations test was carried out on the model. For univariate statistical analysis, we used Student’s *t*-test to obtain *p*-value. Additionally, the receiver operating characteristic (ROC) curve was used for the discovery of biomarkers. For heatmap analysis, data was normalized by the area of internal standard for each lipid species, log-transformed and auto-scaled using Metaboanalyst version 5.0, “http://www.metaboanalyst.ca (accessed on 1 December 2022)”, and data visualization was conducted using Microsoft office Excel 2016. Differences in the biomarkers were compared using Origin, Version 2021 (Origin Lab Corporation, Northampton, MA, USA)

## 3. Results

### 3.1. Lipid Identification

In Tables S2–S11, the retention times, adduct ions, precursor ions, and fragment ions of each lipidome component of PC, PE, plasmenyl-PE, plasmenyl-PC, SM, Cer, lyso-PC, lyso-PE, TG, and DG are listed. The major adduct ions of di- and triacylglycerols tend to have patterns detected as salt adducts, whereas phospholipids and sphingolipids appear predominantly in their protonated state. In this way, the [M + H]^+^, [M + Na]^+^ ions associated with phosphatidylcholine and the [M + NH_4_]^+^, [M + Na]^+^ ions associated with triacylglycerol are listed in the database. Due to the interaction of two fatty acids with the stationary phase, lyso- or monoacyl phospholipids with one fatty acid linked to the glycerol backbone elute sooner than diacyl phospholipids [25]. The chromatographic separation of intra-species is also explained by these phenomena. More retention is achieved with more carbon atoms and fewer double bonds [27]. Triacylglycerols, which are more apolar species, show enhanced retention on the reverse phase column. The diacylglycerols with two fatty acids attached to the glycerol backbone elute earlier compared to the triacylglycerols due to the contribution of three fatty acids to stationary phase interaction in the latter case [33]. In the isocratic portion, resolution between the various triacylglycerol species is accomplished with 100% organic solvent.

Ions were extracted at 5 ppm mass accuracy (from the exact lipid mass), which is sufficient to eliminate the associated isotopes. Lipids were identified by the correlation between retention time, carbon chain length, and degree of unsaturation. LPC 20:4 and LPE 16:0, mono carbon chains, had the fastest elution times at 11.7 min and 12.3 min, respectively (Table S8). Considering the different phospholipid classes, intra-species resolution is readily obtained. There is little doubt that the separation based on fatty acid content predominates the separation based on the head group, as in the case of choline and ethanolamine head groups. Even though choline and ethanolamine have different head groups, PC36:2 (21.5 min) and PE36:2 (22.2 min) almost elute at the similar retention time (Tables S2 and S3). This emphasizes on how crucial MS/MS is to distinguish between these two species. Given their structural similarity, sphingomyelins are frequently found and elute in the vicinity of phospholipids. The sphingomyelins used in our study were distributed between 18.4 min (SM 32:1) and 30.1 min (SM 42:1) (Table S6). Ceramides elute later than their sphingomyelin counterparts due to the absence of the phosphatidylcholine head group. The shortest carbon chain used in the study, Cer 34:1, eluted at 21.9 min and SM 34:1 eluted at 20.2 min (Tables S6 and S7). As mentioned above, di-, triacylglycerol has the latest retention time. TG 60:0, with our method, has a 40.3 min retention time (Table S10). The retention times of each lipid component can be seen in Tables S2–S11.

To verify the obtained database once again, the fragment patterns were confirmed using targeted MS/MS analysis. In the MS/MS spectrum in positive ionization mode, a strong phosphatidylcholine ion at *m*/*z* 184.07 is a marker ion that is produced when protonated phosphatidylcholine lipids dissociate due to collisions [25], confirming that they are PC family lipids (Table S2). Also, for PE family lipids, a spectrum with a neutral loss of 141.05 amu can be generated, which can be clearly identified (Table S3). The plasmalogen does not produce a detectable fragment on its own, but the recognition of the fatty acyl component enables the plasmalogen to be classified as P-16:0 (Tables S4 and S5). SM is difficult to deduce from the MS/MS spectra since fragmentation of the sphingomyelin lipids only gives rise to the phosphorylcholine ion at *m*/*z* 184.07 in positive ionization mode (Table S6). In contrast to the phosphatidylcholine lipids, no carboxylate ion was ever seen. The [M + H]^+^ ion-based MS/MS spectra of ceramide provide a wealth of information (Table S7). In case of DG and TG, A neutral loss of the fatty acids results from running MS/MS on the [M + NH_4_]^+^ ions. TG 48:0 was confirmed of three C16:0 fatty acid glycerol backbone (Tables S10 and S11).

### 3.2. Clinical Characteristics of Participants

**Figure 1:**
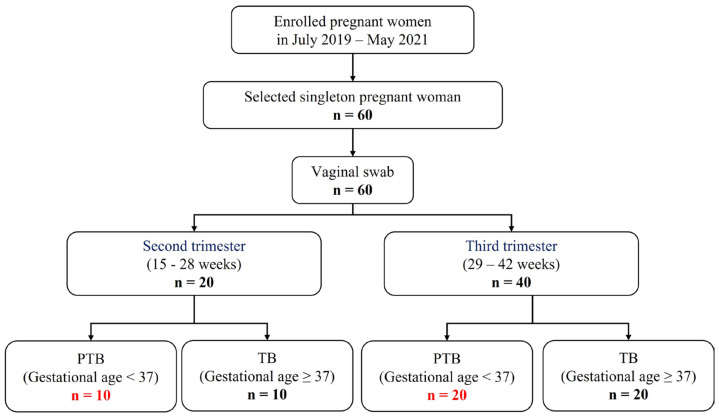
Flow chart of study participants.

A total of 60 individuals were included in this study, which were classified into second or third trimester, according to the sampling week. After that, PTB and TB were divided based on gestational age. As a result, four groups were determined (Figure 1). (Pregnancy 1st trimester: 1–14 weeks, pregnancy 2nd trimester: 15–28 weeks, and pregnancy 3rd trimester: 29–42 weeks). Characteristics with respect to age body mass index (BMI), and race or ethnicity were similar between the groups (Table S1). 

### 3.3. Untargeted Lipid Profiling of CVF between PTB and TB

Total 60 samples (30 PTB and 30 TB) were finally analyzed by the high-throughput lipidomics approach [31,32] to discover altered lipids between PTB and TB conditions. Our high-throughput LC-Q-TOF analysis was capable of profiling 190 lipids including 32 triacylglycerols (TGs), nine diacylglycerols (DGs), 15 sphingomyelins (SMs), nine ceramides (Cers), 29 phosphatidylethanolamines (PEs), 41 phosphatidylcholines (PCs), five lyso-phosphatidylethanolamines (lyso-PEs), seven lyso-phosphatidylcholines (lyso-PCs), 25 plasmenyl-phosphatidylethanolamines (PlsPEs), and 18 plasmenyl-phosphatidylcholines (PlsPCs).

**Figure 2:**
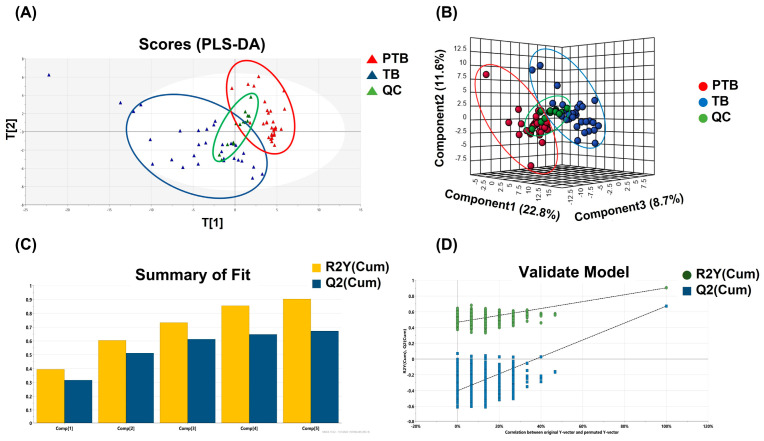
Multivariate statistical analysis. (**A**) PLS-DA score plot and (**B**) 3D PLS-DA score plot of the whole lipidome data between PTB and TB containing QC. (**C**) PLS-DA model revealed the robustness of itself and (**D**) thousand-time permutation test.

Phospholipids and sphingolipids had the most significant alteration between PTB and TB groups. Partial least square-discriminant analysis (PLS-DA) was applied to distinguish between two groups based on whole lipid profiles in Figure 2A. In the score scatter plot, QC samples were strictly clustered between PTB and TB. Figure 2B also display the difference of whole lipidome data for 3D PLS-DA. The predictability was evaluated for the goodness-of-fit (R^2^) of 0.93 and the goodness-of-prediction (Q^2^) of 0.67. These results were shown in Figure 2C. One thousandth-time permutation test indicated an R^2^ of 0.885 and Q^2^ of −6.47 (Figure 2D). Furthermore, principal component analysis, an unsupervised method, was used to verify if PTB and TB exhibit an intra-group lipid variation by sampling trimester (Figure S1). The clusters of second and third trimester samples in both PTB (Figure S1A) and TB (Figure S1B) were not classified substantially in the score plot, indicating that the collected timepoint did not significantly alter the lipid abundances.

**Figure 3:**
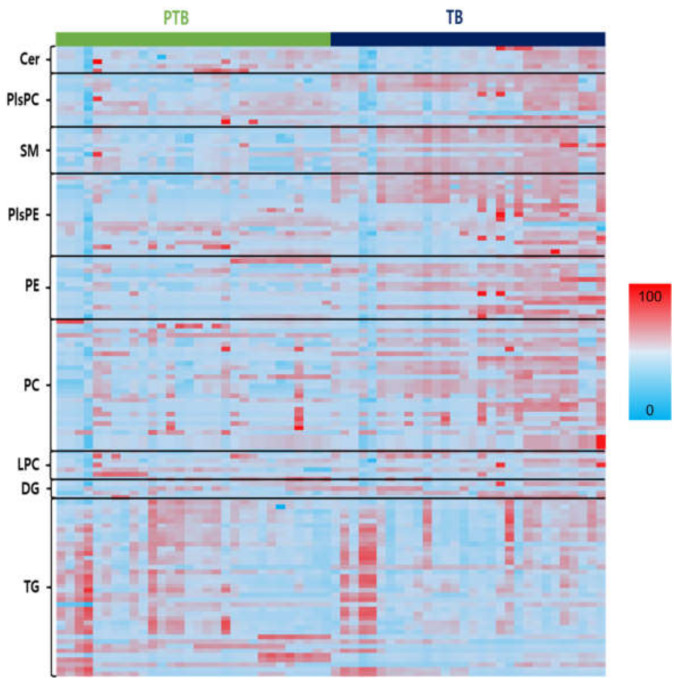
Heats maps representing all lipids between PTB and TB. Normalized expression of each lipid in PTB (n = 30) and TB (n = 30) are shown in scale range from blue to red.

For data exploration, a heatmap of all the lipid species was used to visualize the lipid profiles of PTB and TB in Figure 3. Figure S2A–E show the altered decrease in phospholipids and sphingolipids comparing PTB and TB groups. SMs, PCs, PEs, PlsPCs, and PlsPEs species, which were found different in the two groups in heatmap analysis, decreased significantly in the PTB group, while TGs increased. An overall decreased phospholipid group was seen with significant *p-*value between the PTB and TB (Figures S3–S10). Four PE (C34:0, 36:1, 38:1, and 38:2), five PC (C32:1, 34:1, 36:1, 36:2, and 42:6), four PlsPC (32:0, 34:0, 34:1, and 36:0), and eight PlsPE (C32:0, 32:1, 34:0, 34:1, 36:0, 36:1, 36:2, and 36:3) had *p* < 0.0001. On the other hand, TG with saturated long chain fatty acids were found to be highly abundant in PTB compared to TB (Figures S2–S8).

This result was consistent with the previous study regarding the association between oxidative stress during pregnancy and premature rupture of membranes [34]. Oxidation of these fatty acids, elevated TG levels, and triglyceride synthesis (TGS) can pose serious threats to the body, causing liver and lipid metabolism disorders; pregnancy toxemia is one of the factors that causes disorders during pregnancy [8,35]. Unlike the other lipids, the LPCs and LPEs with one fatty acid had no distinct changes. Notably, SM shows a clear concentration difference between PTB and TB cases (Figure 3). A comparison regarding the lipid species between TB and PTB can be found in Supplementary Information (Figures S3–S10).

### 3.4. Application of Univariate and Multivariate Statis Analysis

**Figure 4:**
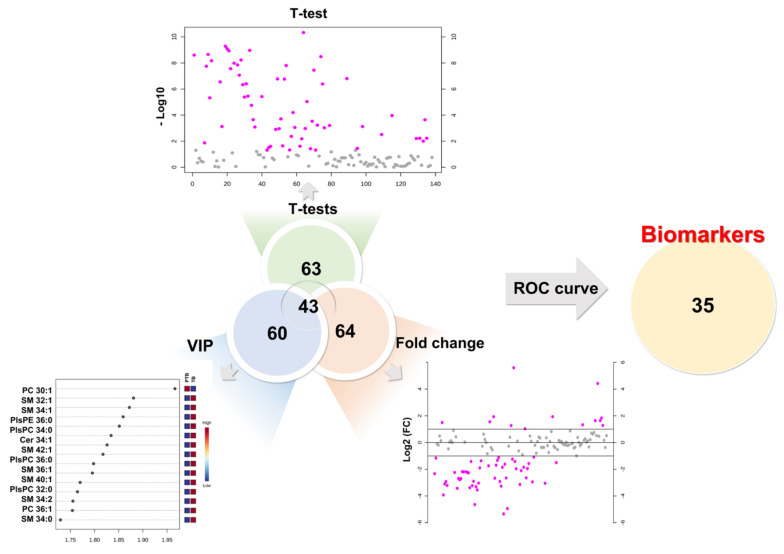
Development of biomarkers. First candidates had to simultaneously satisfy a *p-*value (≤0.05), foldchange (≥2 or ≤0.5), and VIP (≥1). Final biomarkers were applied area under the curve (AUC) of ROC curves (≥0.7).

To determine the diagnostic biomarker, we combined the results of the univariate and multivariate statistical analysis of 190 lipids. As a result of the univariate statistical analysis (*p*-value), a total of 63 lipids had a less than 0.05 *p*-value. Sixty-four lipids were evaluated as more than 2-fold increase or 0.5-fold decrease in PTB conditions. Partial least square-discriminant analysis method measured variable importance for projection (VIP) values of each lipid, while 60 lipids had a value greater than 1. Therefore, we chose lipid biomarker candidates that satisfy three criteria simultaneously, a *p*-value (≤0.05), foldchange (≥2 or ≤0.5), and VIP (≥1). In this process, 43 biomarker candidates were selected (Figure 4).

### 3.5. Evaluation of the Diagnostic Ability of Lipid Biomarkers

**Figure 5:**
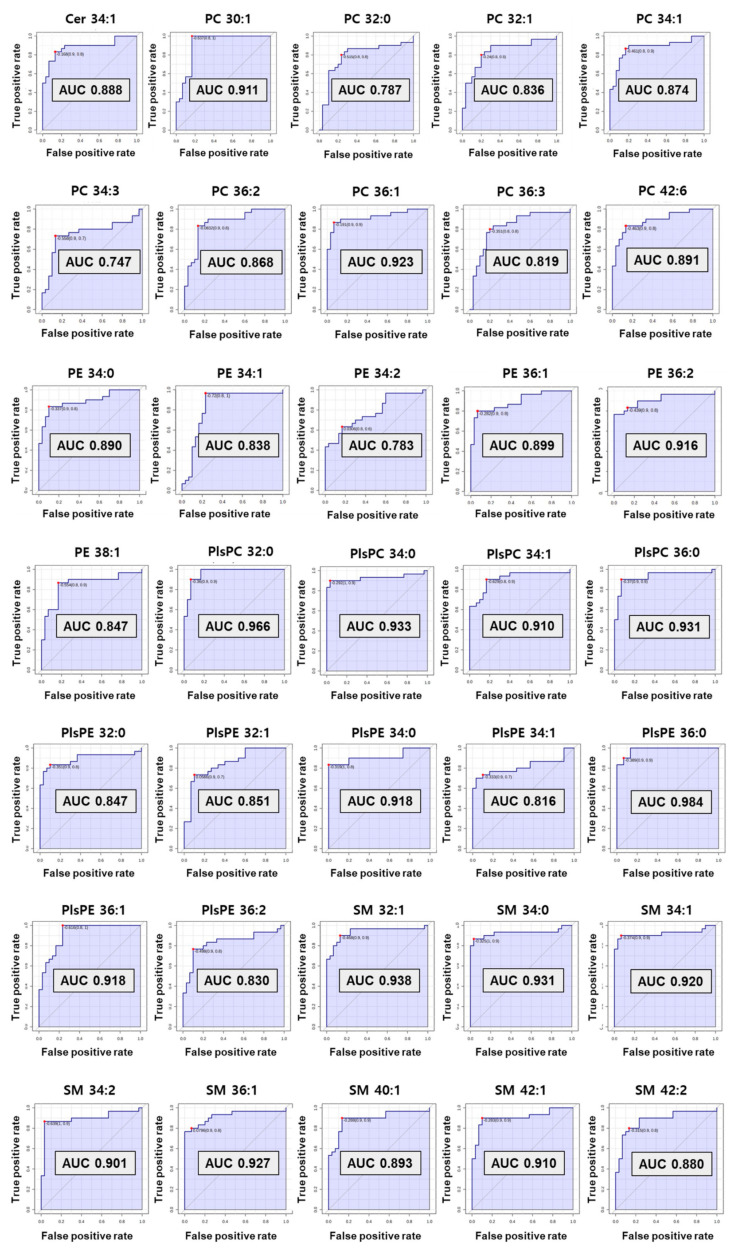
The receiver operating characteristic (ROC) curve of selected biomarkers. AUC values more than 0.7 have potential as biomarker.

The computed area under the ROC curve with the criteria of AUC ≥ 0.7 was applied to decide on the biomarkers with great diagnostic ability (Figure 5). As a result, preferentially eight SMs (C32:1, 34:0, 34:1, 34:2, 36:1, 40:1, 42:1, and 42:2) and one Cer from sphingolipids were selected as diagnostic biomarkers for preterm birth. Among them, six SMs (C32:1, 34:0, 34:1, 34:2, 36:1, and 42:1) showed perfect accuracy in predicting preterm birth, with an AUC of ROC curve greater than 0.9. Cer 34:1 and two SMs (C40:1 and C42:2) had an AUC greater than 0.8.

In phospholipids, nine PCs (C30:1, 32:0, 32:1, 34:1, 34:3, 36:1, 36:2, 36:3, and 42:6) satisfied the criterion of an AUC ≥0.7. A total of six PEs also satisfied the standard AUC. In particular, five PEs (C34:0, 34:1, 36:1, 36:2, and 38:1) indicated perfect accuracy at predicting preterm birth (AUC ≥ 0.8) excluding PE 34:2 (AUC 0.78). In the plasmalogen group, four PlsPCs and seven PlsPEs passed the ROC curve step. All PlsPCs (C32:0, 34:0, 34:1, and 36:0) and three PlsPEs (C34:0, 36:0, and 36:1) had high diagnostic test results with an AUC > 0.9. Four PlsPEs (C32:0, 32:1, 34:1, and 36:2) had an AUC > 0.8. Finally, 35 biomarkers for predicting PTB were selected from the results reflecting the univariate and multivariate results (VIP, *p*-value, and fold change), and ROC curve. The univariate and multivariate outcomes about biomarkers are presented in Table 1.

### 3.6. Comparison New Lipid Biomarkers between PTB and TB

**Figure 6:**
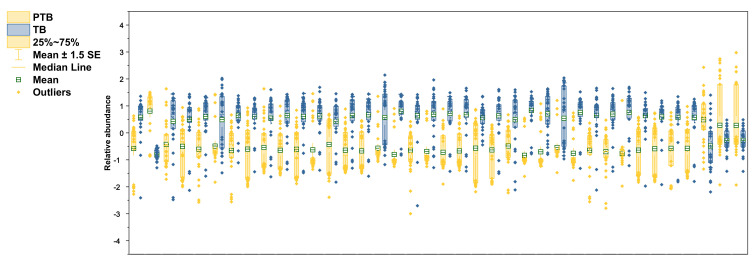
Relative abundance of altered lipidomes in CVF. Yellow color in boxplots indicates PTB groups and blue color indicates TB groups. Boxplots contain the range of 25–75%, median line, mean with S.E.M, and outliers.

The selected lipid biomarkers were significantly reduced in PTB conditions (Figure 6). According to the results of previous research in our laboratory, polar metabolites increase in PTB [28], but most of the lipid biomarkers tend to decrease in PTB. Among the phospholipids selected as biomarkers, not only PCs and PEs, but also PlsPCs and PlsPEs, were decreased in preterm birth. However, only PC 30:1 increased 5.6-fold in the PTB group.

On the other hand, PC 42:6 had decreased by 3.05 times in PTB conditions. In PE group, PE 36:2 had the most decline with 3.26-fold in PTB. The other five PEs (C34:0, 34:1, 34:2, 36:1, and 38:1) also significantly reduce in PTB with less than 1.5-fold. In plasmalogen, PlsPE 36:0 and PlsPC 32:0 had big changes, with 4.64-fold and 3.92-fold decreases, in PTB, respectively.

Remarkably, the reductions were found in most of the SMs detected in PTB, of which the most meaningful decrease was shown as SM 32:1 (log2FC -3.42). Other SMs (C34:0, 34:1, 34:2, 36:1, 40:1, 42:1, and 42:2) represent more than 2-fold declines (−2.70, −2.78, −2.69, −2.19, −2.18, −2.26, and −2.25). In the same pathway, only Cer 34:1 showed a 2.33-fold decrease. Three TGs (C 52:0, 56:0, and 58:0) had *p*-value of less than 0.01 but did not meet the foldchange criterion and were not selected as a biomarker.

## 4. Discussion

We performed an extensive and comprehensive omics study to represent CVF metabolism at the lipidome-level. We successfully identified separate candidates- PCs, PEs, PlsPCs, PlsPE, Cer, and SM as a proof-of-concept. Our findings provided a rich background of knowledge about changes in factors related to lipid metabolism in PTB. These results suggest that dysregulation of the lipids that maintain the cervical lipid membrane and constitute the vaginal microbial composition may be involved in PTB.

**Figure 7:**
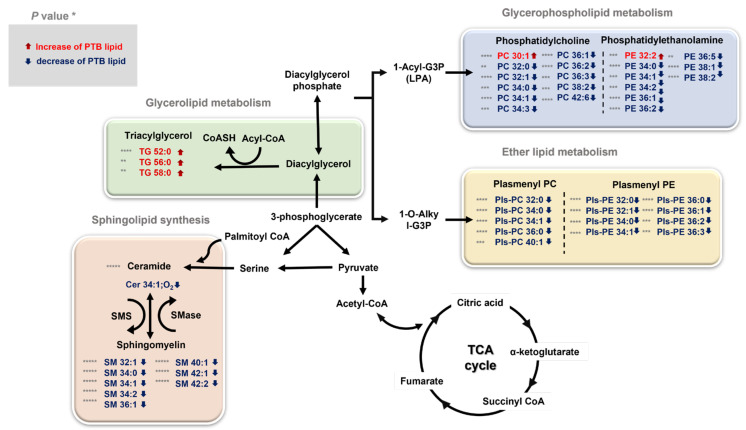
An overview of the integrated metabolic pathways altered by PTB and TB * *p* < 0.05, ** *p* < 0.01, *** *p* < 0.001, and **** *p* < 0.0001. The significantly changed lipids are indicated by arrows: ↓ downregulated and ↑ upregulated in PTBs. TG triglyceride, PC phosphatidylcholine, PE phosphatidylethanolamine, PlsPC plasmenyl-phosphatidylcholine, PlsPE plasmenyl-phosphatidylethanolamine, SM sphingomyelin, Cer Ceramide, TCA cycle tricarboxylic acid cycle, SMS sphingomyelin synthase, SMase sphingomyelin phosphodiesterase.

Our study found significant differences in glycerophospholipids, ether lipids, sphingolipids, and triglycerides, which trigger the most important roles in membrane structure among lipids (Figure 7). There can be a reason for the physiological changes induced by broad lipidome perturbation, which are entailed during pregnancy. For example, maternal adipose tissue is increasingly activated by the anabolism during pregnancy to accumulate and store fatty acid, triglycerides, phospholipids, and cholesterol [36,37]. Particularly, the interaction between the maternal microbiota and fetal environment and the disorder, including PTB, was activated and affected. Therefore, lipid analysis provides a greater insight on lipid metabolites and is potentially useful for defining the biomarkers for disease state such as inflammation or metabolic dysfunctions.

**Figure 8:**
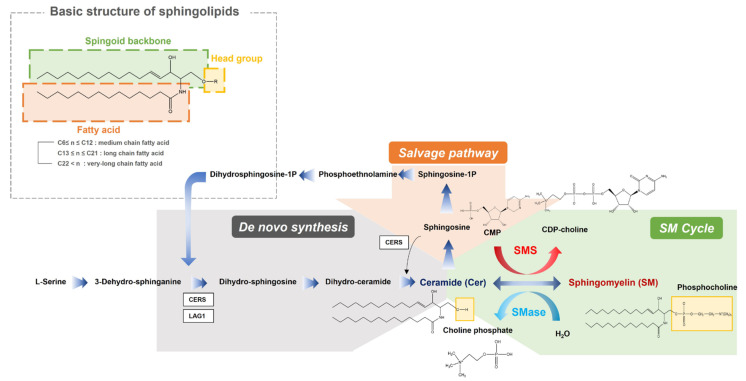
An overview of the integrated metabolic pathways in sphingolipids. SMS sphingomyelin synthase, SMase sphingomyelin phosphodiesterase, CDP-choline cytidine diphosphate-choline, CMP cytidine monophosphate, Sphingosine-1-P sphingosine 1-phosphate.

Specifically, our study investigated the significant downregulation of the anabolic reactions in overall sphingolipid pathway in PTB. Sphingolipids contain a backbone amide attached to a fatty acid residue with a spingoid base and a variable head group (Figure 8). In our result, sphingolipid with long chain (SM 32:1, SM 34:0, SM 34:2 and SM 36:1) and very long chain fatty acids (SM 40:1, SM 42:1 and SM 42:2), out of ten detected sphingomyelins, had significant downregulations in the PTB group, while only one ceramide (Cer 34:1), out of the six detected ceramides, was slightly diminished. These results indicate potential dysfunction of sphingomyelin synthase (SMS), which produces sphingomyelin from ceramides, or upregulation of sphingomyelin phosphodiesterase (SMase). There were many previous studies about potential risks of sphingolipid deficiency in cervicovaginal microenvironment and xenobiotics (microbiome) including their association with a shorter cervix in the second trimester in a high-risk group of PTB [11]. Sphingolipids are present in cell membranes, modify cytoskeletal reorganization and cell adhesion, and alter host immune responses to various pathogens. Especially, this class of lipids organize lipid rafts inside the cell membrane, which makes up cell surface lipids and protein clusters important for cell signaling, motility, growth, and survival. Additional symptoms related to the sphingolipid depletion, including an effect to epidermal permeability during immune response to microbiomes, could lead our findings to the close relationship between a decrease in sphingolipids and preterm birth.

Phospholipids and ether phospholipids (PC, PE, PlsPC, and PlsPE), which were overall downregulated in the PTB group, could be an additional cause of triggering preterm birth. Phospholipids are important constituents of cell membranes and form the building blocks for plasma lipoproteins. Among them, PC and PE were relevantly correlated with obstetric antiphospholipid syndromes (OAPS), which is characterized by the presence of circulating antiphospholipid antibodies (aPL) [38,39].These aPLs target β_2_—glycoprotein I (β_2_—GPI), a circulating blood protein with high affinity that binds to phospholipids present on the cell membrane surface, generating a chain of events that can induce thrombosis. One prior study reported the association of phospholipid reduction and OPAS with PTB [38]. Plasmalogens, which plays a key role as an endogenous antioxidant, can protect other phospholipids and lipoprotein particles from oxidative stress during pregnancy [40,41]. Although plasmalogens are not major phospholipids, according to previous study, levels of plasmalogen were significantly higher in pregnant women than in non-pregnant women [42]. There is also a prior research that higher the level of plasmalogen, longer the average gestation period [8]. Thus, the depletion of plasmalogen affects the pathological outcome. Hence, PlsPE and PlsPC, as well as PC and PE, which are major lipids among phospholipids, should be considered to predict PTB.

These clinical results confirmed changes in various types of lipids in the cervical space. Our observations of metabolic differences between ceramide and sphingomyelin, particularly in sphingolipid biosynthesis and metabolism, providing novel and substantial contributions to the existing literature. Without a doubt, future studies should clarify the contribution of the host and microbial communities to these metabolic changes and ultimately explain the physiological changes that lead to metabolic differences in women with PTB. Investigation of CVF lipid metabolites has great potential for unraveling the mechanisms associated with TB and PTB. In consequence, this study manifests that lipid biomarkers are good predictors of PTB.

## 5. Conclusions

In this study, lipidomics was performed on CVF samples from pregnant women to elucidate changes in lipid metabolism in PTB. A modified folch’s method was used for sample preparation, and high-throughput analysis was performed. We developed our own database using the unique characteristics of lipid and conducted data processing and statistical analysis. As a result, there were significant changes in metabolisms related to phospholipids and sphingolipids in pregnant women who experienced TB and PTB, and 35 lipid biomarkers related thereto were developed. Thereby, we discovered the influence of not only PC and PE, the major phospholipids, but also plasmalogen in PTB. In sphingolipids, it was emphasized that the deficiency of sphingomyelin due to a problem with the synthesis process is an important key in predicting premature birth. Therefore, the differences in lipid composition of CVF metabolites between PTB and TB reveal major metabolic pathways that may support PTB, making this study importantly informative for predicting the pregnancy state.

## Figures and Tables

**Table 1:** Variable importance for projection value (VIP value), *t*-test, and fold change (FC = PTB/TB) of developed biomarkers.

Compounds	*p-*Value	log2 (FC)	VIP
Cer 34:1; O2	1.56 × 10^−8^	−2.33	1.95
PC 30:1	4.60 × 10^−11^	5.60	2.00
PC 32:0	1.06 × 10^−3^	−1.42	1.14
PC 32:1	9.12 × 10^−6^	−1.96	1.48
PC 34:1	3.53 × 10^−8^	−2.10	1.76
PC 34:3	5.91 × 10^−4^	−1.85	1.19
PC 36:1	3.25×10^−9^	−2.64	1.86
PC 36:2	4.04 × 10^−7^	−1.95	1.65
PC 36:3	9.32 × 10^−4^	−1.85	1.15
PC 42:6	1.57 × 10^−7^	−3.05	1.70
PE 34:0	1.66 × 10^−7^	−2.67	1.69
PE 34:1	1.10 × 10^−3^	−1.69	1.13
PE 34:2	1.96 × 10^−4^	−2.69	1.27
PE 36:1	1.72 × 10^−7^	−2.92	1.69
PE 36:2	1.57 × 10^−8^	−3.26	1.80
PE 38:1	6.35 × 10^−5^	−2.65	1.36
plasmenyl-PC 32:0	1.37 × 10^−7^	−3.92	1.88
plasmenyl-PC 34:0	1.38 × 10^−8^	−3.10	1.97
plasmenyl-PC 34:1	2.65 × 10^−5^	−2.92	1.57
plasmenyl-PC 36:0	7.80 × 10^−9^	−3.21	1.90
plasmenyl-PE 32:0	4.42 × 10^−6^	−3.39	1.72
plasmenyl-PE 32:1	3.41 × 10^−6^	−1.41	1.59
plasmenyl-PE 34:0	3.03 × 10^−6^	−3.23	1.74
plasmenyl-PE 34:1	8.53 × 10^−6^	−2.90	1.61
plasmenyl-PE 36:0	7.86 × 10^−9^	−4.64	1.98
plasmenyl-PE 36:1	1.72 × 10^−5^	−3.31	1.51
plasmenyl-PE 36:2	2.09 × 10^−4^	−3.55	1.32
SM 32:1	2.36 × 10^−9^	−3.42	1.99
SM 34:0	3.47 × 10^−7^	−2.70	1.86
SM 34:1	1.42 × 10^−8^	−2.78	1.99
SM 34:2	1.26 × 10^−9^	−2.69	1.88
SM 36:1	7.43 × 10^−8^	−2.19	1.90
SM 40:1	3.27 × 10^−8^	−2.18	1.89
SM 42:1	3.15 × 10^−8^	−2.26	1.92
SM 42:2	9.90 × 10^−8^	−2.25	1.81

## Data Availability

The data presented in this study are available on request from the corresponding author. The data are not publicly available due to ethical reasons.

## Supplementary Materials

The following supporting information can be downloaded at: https://www.mdpi.com/article/10.3390/metabo13020177/s1, Figure S1: PCA score plots of PTB (second trimester, n = 10; third trimester, n = 20) and TB (second trimester, n = 10; third trimester, n = 20), Figure S2: Heats maps representing (A) PlsPCs and PlsPEs, (B) PEs, (C) Cers, (D) SMs, (E) PCs between PTB and TB., Figure S3: Log 10 peak area of lipidomes in PCs. Orange color in bar graphs and asterisk indicates PTB groups and green color indicates TB. Bar graphs are mean with * *p* < 0.05, ** *p* < 0.01, *** *p* < 0.001, and **** 0.0001. These are same in Figures S3–S10, Figure S4: Log 10 peak area of lipidomes in PEs, Figure S5: Log 10 peak area of lipidomes in PlsPCs, Figure S6: Log 10 peak area of lipidomes in PlsPEs, Figure S7: Log 10 peak area of lipidomes in SMs, Figure S8: Log 10 peak area of lipidomes in Cers, Figure S9: Log 10 peak area of lipidomes in TGs, Figure S10: Log 10 peak area of lipidomes in LPCs; Table S1: Descriptive characteristics among participants, Table S2: List of identified lipidomes in PCs, Table S3: List of identified lipidomes in PEs, Table S4: List of identified lipidomes in plasmenyl-PEs, Table S5: List of identified lipidomes in plasmenyl-PCs, Table S6: List of identified lipidomes in SMs, Table S7: List of identified lipidomes in Cers, Table S8: List of identified lipidomes in lyso-PCs, Table S9: List of identified lipidomes in lyso-PEs, Table S10: List of identified lipidomes in TGs, Table S11: List of identified lipidomes in DGs.

## References

[1] Goldenberg R.L., Culhane J.F., Iams J.D., Romero R. (2008). Epidemiology and causes of preterm birth. Lancet.

[2] Crump C. (2020). Preterm birth and mortality in adulthood: A systematic review. J. Perinatol..

[3] Walani S.R. (2020). Global burden of preterm birth. Int. J. Gynecol. Obstet..

[4] Kaplan Z.A.O., Ozgu-Erdinc A.S. (2018). Prediction of preterm birth: Maternal characteristics, ultrasound markers, and biomarkers: An updated overview. J. Pregnancy.

[5] Graça G., Diaz S.O., Pinto J., Barros A.S., Duarte I.F., Goodfellow B.J., Galhano E., Pita C., Almeida M.d.C., Carreira I.M. (2012). Can Biofluids Metabolic Profiling Help to Improve Healthcare during Pregnancy?. Spectrosc. Int. J..

[6] Casas M., Valvi D., Luque N., Ballesteros-Gomez A., Carsin A.E., Fernandez M.F., Koch H.M., Mendez M.A., Sunyer J., Rubio S. (2013). Dietary and sociodemographic determinants of bisphenol A urine concentrations in pregnant women and children. Env. Int.

[7] Needham L.L., Sexton K. (2000). Introduction and overview: Assessing children’s exposure to hazardous environmental chemicals: An overview of selected research challenges and complexities. J. Expo. Sci. Environ. Epidemiol..

[8] Bandyopadhyay S., Dasgupta D., Sarkar N., Chakraborty M. (2021). A Review on Current scenario of Lipid Metabolic Disorders. Int. J. Pharm. Biol. Sci..

[9] Behrman R.E., Butler A.S. (2007). Preterm Birth: Causes, Consequences, and Prevention.

[10] Bewick V., Cheek L., Ball J. (2004). Statistics review 13: Receiver operating characteristic curves. Crit. Care.

[11] Gerson K.D., Yang N., Anton L., Levy M., Ravel J., Elovitz M.A., Burris H.H. (2022). Second trimester short cervix is associated with decreased abundance of cervicovaginal lipid metabolites. Am. J. Obs. Gynecol.

[12] Ghartey J., Bastek J.A., Brown A.G., Anglim L., Elovitz M.A. (2015). Women with preterm birth have a distinct cervicovaginal metabolome. Am. J. Obs. Gynecol..

[13] Burris H.H., Gerson K.D., Woodward A., Redhunt A., Ledyard R., Brennan K., Baccarelli A.A., Hecht J.L., Collier A.Y., Hacker M.R. (2023). Cervical microRNA expression and spontaneous preterm birth. Am. J. Obs. Gynecol MFM.

[14] Ansari A., Lee H., You Y.-A., Jung Y., Park S., Kim S.M., Hwang G.-S., Kim Y.J. (2020). Identification of Potential Biomarkers in the Cervicovaginal Fluid by Metabolic Profiling for Preterm Birth. Metabolites.

[15] Catov J.M., Bodnar L.M., Kip K.E., Hubel C., Ness R.B., Harger G., Roberts J.M. (2007). Early pregnancy lipid concentrations and spontaneous preterm birth. Am. J. Obstet. Gynecol..

[16] Cinque B., Di Marzio L., Centi C., Di Rocco C., Riccardi C., Cifone M.G. (2003). Sphingolipids and the immune system. Pharmacol. Res..

[17] Cox S., King M., Casey M., MacDonald P. (1993). Interleukin-1 beta,-1 alpha, and-6 and prostaglandins in vaginal/cervical fluids of pregnant women before and during labor. J. Clin. Endocrinol. Metab..

[18] Wang C., Zhu W., Wei Y., Su R., Feng H., Hadar E., Hod M., Yang H. (2017). The associations between early pregnancy lipid profiles and pregnancy outcomes. J. Perinatol..

[19] Herrera E. (2002). Lipid metabolism in pregnancy and its consequences in the fetus and newborn. Endocrine.

[20] Nagle J.F., Tristram-Nagle S. (2000). Structure of lipid bilayers. Biochim. Biophys. Acta (BBA)-Rev. Biomembr..

[21] Babayan V.K. (1987). Medium chain triglycerides and structured lipids. Lipids.

[22] Dawson R. (1957). The animal phospholipids: Their structure, metabolism and biological significance. Biol. Rev..

[23] Fakhr Y., Brindley D.N., Hemmings D.G. (2021). Physiological and pathological functions of sphingolipids in pregnancy. Cell. Signal..

[24] Mizugishi K., Inoue T., Hatayama H., Bielawski J., Pierce J.S., Sato Y., Takaori-Kondo A., Konishi I., Yamashita K. (2015). Sphingolipid pathway regulates innate immune responses at the fetomaternal interface during pregnancy. J. Biol. Chem..

[25] Sandra K., Pereira Ados S., Vanhoenacker G., David F., Sandra P. (2010). Comprehensive blood plasma lipidomics by liquid chromatography/quadrupole time-of-flight mass spectrometry. J. Chromatogr. A.

[26] Yan L., Han P., Man J., Tian Y., Wang F., Wang J. (2021). Discovery of lipid profiles of type 2 diabetes associated with hyperlipidemia using untargeted UPLC Q-TOF/MS-based lipidomics approach. Clin. Chim. Acta.

[27] t’Kindt R., Jorge L., Dumont E., Couturon P., David F., Sandra P., Sandra K. (2012). Profiling and characterizing skin ceramides using reversed-phase liquid chromatography-quadrupole time-of-flight mass spectrometry. Anal. Chem..

[28] Lee J.Y., Seo S., Shin B., Hong S.H., Kwon E., Park S., Hur Y.M., Lee D.K., Kim Y.J., Han S.B. (2022). Development of a New Biomarker Model for Predicting Preterm Birth in Cervicovaginal Fluid. Metabolites.

[29] Zhao Q., Ma Z., Wang X., Liang M., Wang W., Su F., Yang H., Gao Y., Ren Y. (2020). Lipidomic biomarkers of extracellular vesicles for the prediction of preterm birth in the early second trimester. J. Proteome Res..

[30] Sarafian M.H., Gaudin M., Lewis M.R., Martin F.-P., Holmes E., Nicholson J.K., Dumas M.-E. (2014). Objective set of criteria for optimization of sample preparation procedures for ultra-high throughput untargeted blood plasma lipid profiling by ultra performance liquid chromatography–mass spectrometry. Anal. Chem..

[31] Lee D.K., Long N.P., Jung J., Kim T.J., Na E., Kang Y.P., Kwon S.W., Jang J. (2019). Integrative lipidomic and transcriptomic analysis of X-linked adrenoleukodystrophy reveals distinct lipidome signatures between adrenomyeloneuropathy and childhood cerebral adrenoleukodystrophy. Biochem. Biophys. Res. Commun..

[32] Welti R., Wang X. (2004). Lipid species profiling: A high-throughput approach to identify lipid compositional changes and determine the function of genes involved in lipid metabolism and signaling. Curr. Opin. Plant Biol..

[33] Lin J.-T., Woodruff C.L., McKeon T.A. (1997). Non-aqueous reversed-phase high-performance liquid chromatography of synthetic triacylglycerols and diacylglycerols. J. Chromatogr. A.

[34] Longini M., Perrone S., Vezzosi P., Marzocchi B., Kenanidis A., Centini G., Rosignoli L., Buonocore G. (2007). Association between oxidative stress in pregnancy and preterm premature rupture of membranes. Clin. Biochem..

[35] Xue Y., Guo C., Hu F., Sun D., Liu J., Mao S. (2019). Molecular mechanisms of lipid metabolism disorder in livers of ewes with pregnancy toxemia. Animal.

[36] Herrera E., Ortega-Senovilla H. (2010). Maternal lipid metabolism during normal pregnancy and its implications to fetal development. Clin. Lipidol..

[37] Herrera E., Ortega-Senovilla H. (2014). Lipid metabolism during pregnancy and its implications for fetal growth. Curr. Pharm. Biotechnol..

[38] Morillon A.-C., Yakkundi S., Thomas G., Gethings L.A., Langridge J.I., Baker P.N., Kenny L.C., English J.A., McCarthy F.P. (2020). Association between phospholipid metabolism in plasma and spontaneous preterm birth: A discovery lipidomic analysis in the cork pregnancy cohort. Metabolomics.

[39] Högdén A., Antovic A., Berg E., Bremme K., Chaireti R. (2019). Obstetric outcomes in patients with primary thrombotic and obstetric antiphospholipid syndrome and its relation to the antiphospholipid antibody profile. Lupus.

[40] Reiss D., Beyer K., Engelmann B. (1997). Delayed oxidative degradation of polyunsaturated diacyl phospholipids in the presence of plasmalogen phospholipids in vitro. Biochem. J..

[41] Engelmann B., Brautigam C., Thiery J. (1994). Plasmalogen phospholipids as potential protectors against lipid peroxidation of low-density lipoproteins. Biochem. Biophys. Res. Commun..

[42] Kerstell J., Svanborg A., Vikrot O. (1967). Plasmalogens in Human Plasma During Pregnancy: A Study of Healthy Non-pregnant and Pregnant Women. Acta Med. Scand..

