# Supplementary material for: Lipidomic Analysis of Cervicovaginal Fluid for Elucidating Prognostic Biomarkers and Relevant Phospholipid and Sphingolipid Pathways in Preterm Birth
Source: Metabolites. 2023 Jan 25;13(2):177. doi: 10.3390/metabo13020177 (PMC9963162; doi:10.3390/metabo13020177)

Supplementary information

Lipidomic analysis of cervicovaginal fluid for elucidating prognostic biomarkers and relevant phospholipid and sphingolipid pathways in preterm birth

Se Hee Hong[1], Ji-Youn Lee[1], Sumin Seo[1], Bohyun Shin[1], Cho Hee Jeong[1], Eunbin Bae[1], Jiyu Kim[1], Donghee Lee[1], Byungchan An[1], Minki Shim[1], Jung Hoon Shin[1], Young Ju Kim[2], Dong-Kyu Lee[1,*], and Sang Beom Han[1,*]

[1]College of Pharmacy, Chung-Ang University, 84 Heukseok-ro, Dongjak-gu, Seoul 06974, Korea

[2]Department of Obstetrics and Gynecology and Ewha Medical Research Institute, College of Medicine, Ewha Womans University, Seoul 07984, Korea

* Correspondence: leedk@cau.ac.kr (D.-K.L.); hansb@cau.ac.kr (S.B.H.)

Table of Contents

Supplementary Tables **3**

Table S1. Descriptive characteristics among participants 3

Table S2. List of identified lipidomes in PCs 6

Table S3. List of identified lipidomes in PEs 7

Table S4. List of identified lipidomes in Plasmenyl-PEs 8

Table S5. List of identified lipidomes in Plasmenyl-PCs 9

Table S6. List of identified lipidomes in SMs 10

Table S7. List of identified lipidomes in Cers 11

Table S8. List of identified lipidomes in LysoPCs 12

Table S9. List of identified lipidomes in LysoPEs 12

Table S10. List of identified lipidomes in TGs 13

Table S11. List of identified lipidomes in DGs 15

Supplementary Figures **16**

Figure S1. Spearman rank correlation of lipids in CVF which were used in our study between PTB and TB 16

Figure S2. Heats maps representing (A) PlsPCs and PlsPEs, (B) PEs, (C) Cers, (D) SMs, (E) PCs between PTB and TB. 17

Figure S3. Log 10 peak area of lipidomes in PCs. Orange color in bar graphs and asterisk indicates PTB groups and green color indicates TB. Bar graphs are mean with *p<0.05, **p<0.01, ***p<0.001, and ****0.0001. These are same in Figure S3-S10 18

Figure S4. Log 10 peak area of lipidomes in PEs 19

Figure S5. Log 10 peak area of lipidomes in PlsPCs 20

Figure S6. Log 10 peak area of lipidomes in PlsPEs 21

Figure S7. Log 10 peak area of lipidomes in SMs 22

Figure S8. Log 10 peak area of lipidomes in Cers 23

Figure S9. Log 10 peak area of lipidomes in TGs 24

Figure S10. Log 10 peak area of lipidomes in LPCs 25

Supplementary Tables

Table S1. Descriptive characteristics among participants with (A) PTB and (B) TB

(A)

No.	Age (y)	Gestational age (wk.)	Sampling week	Pre-Pregnancy BMI (kg/m^2)
P1	27	34.6	34.5	20.24
P2	33	33.2	33.2	16.57
P3	36	36.3	19.2	21.23
P4	30	36.2	19.1	24.30
P5	27	32.3	31.6	23.11
P6	30	36.0	18.1	19.59
P7	25	33.2	33.2	19.95
P8	34	34.0	34.0	20.43
P9	36	32.2	32.2	21.95
P10	37	36.3	33.5	21.23
P11	33	32.5	32.4	25.24
P12	30	36.1	24.1	23.51
P13	26	28.2	28.2	27.10
P14	36	31.2	31.1	31.69
P15	36	20.6	20.4	22.66
P16	33	36.0	35.1	21.20
P17	30	36.1	36.0	23.51
P18	31	36.4	36.3	21.80
P19	37	36.1	35.5	22.64
P20	30	30.6	30.6	21.88
P21	40	36.6	34.4	27.67
P22	39	27.2	26.4	25.33
P23	28	36.1	36.0	21.84
P24	34	35.1	33.3	21.63
P25	27	33.5	33.4	20.06
P26	31	25.4	23.4	20.75
P27	26	31.6	29.3	21.72
P28	30	31.0	30.6	21.38

P29	36	33.2	21.6	21.45
P30	30	32.6	26.1	17.04

(B)

No. -	Age (y)	Gestational age (wk.)	Sampling week	Pre-Pregnancy BMI (kg/m^2)
T1	37	39.6	37	19.03
T2	35	39.6	37.1	20.49
T3	28	40	37.2	18.83
T4	29	39.4	37.1	21.16
T5	34	38.4	35.5	21.49
T6	29	39.2	36.1	21.13
T7	34	39.3	36.3	21.2
T8	32	40.4	39.2	22.02
T9	35	40.3	21	21.76
T10	32	38.1	23.6	22.12
T11	40	38.4	20.3	20.96
T12	36	38.3	21.6	20.72
T13	31	40.5	37.3	17.15
T14	33	39.3	24.2	18.65
T15	31	40.1	37.5	17.34
T16	28	37.2	36.1	20.7
T17	37	38.3	18	23.09
T18	37	37.6	21.3	20.63
T19	21	38.6	37.1	23.95
T20	30	37.4	36.3	20.69
T21	33	37.6	37.2	19.71
T22	39	38.6	34.3	21.1
T23	38	37.6	36.3	20.63
T24	35	39.5	24	21.23
T25	36	37.6	21.3	24.67
T26	30	38.3	36.3	26.4
T27	31	39	29.6	20.03
T28	33	39.3	39.2	19.1
T29	34	37.6	37.5	25.85
T30	35	39.3	24	20.45

Table S2. List of identified lipidomes in PCs

Identity	Retention time (min)	Adduct ion	Precursor ion (m/z)	Fragment ion (m/z)
PC 20:0	13.2	$[M+H]^+$	566.385	184.073
PC 28:0	18.1	$[M+H]^+$	678.507	184.073
PC 30:0	19.8	$[M+H]^+$	706.539	184.073
PC 30:1	18.1	$[M+H]^+$	704.523	184.073
PC 32:0	21.9	$[M+H]^+$	734.570	184.073
PC 32:1	19.9	$[M+H]^+$	732.554	184.073
PC 32:2	18.5	$[M+H]^+$	730.539	184.073
PC 32:3	17.8	$[M+H]^+$	728.523	184.073
PC 34:0	24.1	$[M+H]^+$	762.601	184.073
PC 34:1	21.9	$[M+H]^+$	760.586	184.073
PC 34:2	20.2	$[M+H]^+$	758.570	184.073
PC 34:3	19.0	$[M+H]^+$	756.554	184.073
PC 34:4	17.9	$[M+H]^+$	754.539	184.073
PC 36:0	26.7	$[M+H]^+$	790.633	184.073
PC 36:1	24.0	$[M+H]^+$	788.617	184.073
PC 36:2	21.5	$[M+H]^+$	786.601	184.073
PC 36:3	20.9	$[M+H]^+$	784.586	184.073
PC 36:4	19.5	$[M+H]^+$	782.570	184.073
PC 36:5	17.9	$[M+H]^+$	780.554	184.073
PC 38:1	26.2	$[M+H]^+$	816.648	184.073
PC 38:2	23.6	$[M+H]^+$	814.633	184.073
PC 38:3	22.4	$[M+H]^+$	812.617	184.073
PC 38:4	21.3	$[M+H]^+$	810.601	184.073
PC 38:5	19.4	$[M+H]^+$	808.586	184.073
PC 38:6	18.8	$[M+H]^+$	806.570	184.073
PC 38:7	17.4	$[M+H]^+$	804.554	184.073
PC 40:1	29.2	$[M+H]^+$	844.680	184.073
PC 40:2	26.3	$[M+H]^+$	842.664	184.073
PC 40:3	24.3	$[M+H]^+$	840.648	184.073
PC 40:4	22.3	$[M+H]^+$	838.633	184.073
PC 40:5	21.2	$[M+H]^+$	836.617	184.073
PC 40:6	19.3	$[M+H]^+$	834.601	184.073
PC 40:7	18.8	$[M+H]^+$	832.586	184.073
PC 40:8	17.7	$[M+H]^+$	830.570	184.073
PC 42:1	31.0	$[M+H]^+$	872.711	184.073
PC 42:2	28.7	$[M+H]^+$	870.695	184.073
PC 42:3	26.1	$[M+H]^+$	868.680	184.073
PC 42:4	24.1	$[M+H]^+$	866.664	184.073
PC 42:5	23.3	$[M+H]^+$	864.648	184.073
PC 42:6	21.1	$[M+H]^+$	862.633	184.073
PC 42:7	19.6	$[M+H]^+$	860.617	184.073

Table S3. List of identified lipidomes in PEs

Identity	Retention time (min)	Adduct ion	Precursor ion (m/z)	Fragment ion (m/z)
PE 20:0	13.4	$[M+H]^+$	524.337	383.337
PE 32:0	22.5	$[M+H]^+$	692.523	551.509
PE 32:1	20.2	$[M+H]^+$	690.507	549.487
PE 32:2	18.5	$[M+H]^+$	688.492	547.471
PE 34:0	24.8	$[M+H]^+$	720.554	579.532
PE 34:1	22.3	$[M+H]^+$	718.539	577.522
PE 34:2	20.1	$[M+H]^+$	716.523	575.503
PE 34:3	18.7	$[M+H]^+$	714.507	573.457
PE 36:1	24.5	$[M+H]^+$	746.570	605.554
PE 36:2	22.2	$[M+H]^+$	744.554	603.533
PE 36:3	20.4	$[M+H]^+$	742.539	601.518
PE 36:4	19.9	$[M+H]^+$	740.523	599.503
PE 36:5	18.3	$[M+H]^+$	738.507	597.490
PE 38:1	27.1	$[M+H]^+$	774.601	633.579
PE 38:2	24.3	$[M+H]^+$	772.586	631.554
PE 38:3	23.7	$[M+H]^+$	770.570	629.548
PE 38:4	21.9	$[M+H]^+$	768.554	627.533
PE 38:5	19.8	$[M+H]^+$	766.539	625.517
PE 38:7	17.8	$[M+H]^+$	762.507	621.488
PE 40:10	17.8	$[M+H]^+$	784.492	643.464
PE 40:2	26.8	$[M+H]^+$	800.617	659.581
PE 40:3	25.5	$[M+H]^+$	798.601	657.580
PE 40:4	23.4	$[M+H]^+$	796.586	655.570
PE 40:5	22.6	$[M+H]^+$	794.570	653.256
PE 40:6	21.0	$[M+H]^+$	792.554	651.533
PE 40:7	19.2	$[M+H]^+$	790.539	649.519
PE 42:10	19.2	$[M+H]^+$	812.523	671.498
PE 42:6	22.2	$[M+H]^+$	820.586	679.567
PE 42:7	20.2	$[M+H]^+$	818.570	677.546

Table S4. List of identified lipidomes in Plasmenyl-PEs

Identity	Retention time (min)	Adduct ion	Precursor ion (m/z)	Fragment ion (m/z)
plasmenyl-PE 32:0	23.6	$[M+H]^+$	676.528	535.508
plasmenyl-PE 32:1	21.2	$[M+H]^+$	674.512	533.492
plasmenyl-PE 34:0	26.2	$[M+H]^+$	704.559	563.536
plasmenyl-PE 34:1	23.5	$[M+H]^+$	702.544	561.518
plasmenyl-PE 36:0	26.5	$[M+H]^+$	732.591	591.557
plasmenyl-PE 36:1	26.0	$[M+H]^+$	730.575	589.552
plasmenyl-PE 36:2	23.3	$[M+H]^+$	728.559	587.538
plasmenyl-PE 36:3	22.6	$[M+H]^+$	726.544	585.518
plasmenyl-PE 36:4	20.8	$[M+H]^+$	724.528	583.508
plasmenyl-PE 38:1	28.6	$[M+H]^+$	758.606	617.579
plasmenyl-PE 38:2	25.5	$[M+H]^+$	756.591	615.569
plasmenyl-PE 38:3	25.0	$[M+H]^+$	754.575	613.544
plasmenyl-PE 38:4	23.0	$[M+H]^+$	752.559	611.537
plasmenyl-PE 38:5	20.7	$[M+H]^+$	750.544	609.522
plasmenyl-PE 38:6	20.0	$[M+H]^+$	748.528	607.505
plasmenyl-PE 40:1	30.8	$[M+H]^+$	786.638	645.619
plasmenyl-PE 40:3	26.8	$[M+H]^+$	782.606	641.584
plasmenyl-PE 40:4	24.1	$[M+H]^+$	780.591	639.567
plasmenyl-PE 40:5	22.8	$[M+H]^+$	778.575	637.556
plasmenyl-PE 40:6	22.0	$[M+H]^+$	776.559	635.538
plasmenyl-PE 42:2	29.7	$[M+H]^+$	812.653	671.638
plasmenyl-PE 42:3	29.0	$[M+H]^+$	810.638	669.627
plasmenyl-PE 42:4	26.6	$[M+H]^+$	808.622	667.597
plasmenyl-PE 42:5	25.1	$[M+H]^+$	806.606	665.586
plasmenyl-PE 42:6	22.6	$[M+H]^+$	804.591	663.573

Table S5. List of identified lipidomes in Plasmenyl-PCs

Identity	Retention time (min)	Adduct ion	Precursor ion (m/z)	Fragment ion (m/z)
plasmenyl-PC 30:0	19.1	$[M+H]^+$	690.544	184.073
plasmenyl-PC 32:0	20.9	$[M+H]^+$	718.575	184.073
plasmenyl-PC 34:0	23.1	$[M+H]^+$	746.606	184.073
plasmenyl-PC 34:1	22.7	$[M+H]^+$	744.591	184.073
plasmenyl-PC 36:0	25.7	$[M+H]^+$	774.638	184.073
plasmenyl-PC 36:1	25.2	$[M+H]^+$	772.622	184.073
plasmenyl-PC 36:2	22.6	$[M+H]^+$	770.606	184.073
plasmenyl-PC 36:3	20.6	$[M+H]^+$	768.591	184.073
plasmenyl-PC 36:4	20.5	$[M+H]^+$	766.575	184.073
plasmenyl-PC 36:5	20.2	$[M+H]^+$	764.559	184.073
plasmenyl-PC 38:1	27.3	$[M+H]^+$	800.653	184.073
plasmenyl-PC 38:3	22.1	$[M+H]^+$	796.622	184.073
plasmenyl-PC 38:4	20.5	$[M+H]^+$	794.606	184.073
plasmenyl-PC 38:5	20.2	$[M+H]^+$	792.591	184.073
plasmenyl-PC 40:1	29.3	$[M+H]^+$	828.685	184.073
plasmenyl-PC 40:2	26.7	$[M+H]^+$	826.669	184.073
plasmenyl-PC 40:4	27.3	$[M+H]^+$	822.638	184.073
plasmenyl-PC 42:5	26.8	$[M+H]^+$	848.653	184.073

Table S6. List of identified lipidomes in SMs

Identity	Retention time (min)	Adduct ion	Precursor ion (m/z)	Fragment ion (m/z)
SM 32:1	18.4	$[M+H]^+$	675.544	184.073
SM 34:2	18.4	$[M+H]^+$	701.560	184.073
SM 34:1	20.2	$[M+H]^+$	703.575	184.073
SM 34:0	21.0	$[M+H]^+$	705.591	184.073
SM 35:1	21.4	$[M+H]^+$	717.593	184.073
SM 36:2	20.2	$[M+H]^+$	729.591	184.073
SM 36:1	22.3	$[M+H]^+$	731.607	184.073
SM 36:0	23.4	$[M+H]^+$	733.622	184.073
SM 38:1	24.3	$[M+H]^+$	759.638	184.073
SM 38:0	26.0	$[M+H]^+$	761.654	184.073
SM 40:2	24.4	$[M+H]^+$	785.654	184.073
SM 40:1	27.6	$[M+H]^+$	787.669	184.073
SM 40:0	28.8	$[M+H]^+$	789.685	184.073
SM 42:2	27.0	$[M+H]^+$	813.685	184.073
SM 42:1	30.1	$[M+H]^+$	815.701	184.073

Table S7. List of identified lipidomes in Cers

Identity	Retention time (min)	Adduct ion	Precursor ion (m/z)	Fragment ion (m/z)
Cer 34:1; O2	21.9	$[M+H]^+$	538.519	264.267
Cer 35:1; O2	23.3	$[M+H]^+$	552.535	264.267
Cer 36:1; O2	24.2	$[M+H]^+$	566.551	264.267
Cer 38:1; O2	27.0	$[M+H]^+$	594.582	264.267
Cer 40:1; O2	29.6	$[M+H]^+$	622.613	264.267
Cer 40:2; O2	26.4	$[M+H]^+$	620.598	264.267
Cer 42:1; O2	31.4	$[M+H]^+$	650.645	264.267
Cer 42:2; O2	29.2	$[M+H]^+$	648.629	264.267
Cer 44:2; O2	30.5	$[M+H]^+$	676.660	264.267

Table S8. List of identified lipidomes in LysoPCs

Identity	Retention time (min)	Adduct ion	Precursor ion (m/z)	Fragment ion (m/z)
LysoPC 16:0	12.3	$[M+H]^+$	496.340	184.073
LysoPC 18:0	14.1	$[M+H]^+$	524.372	184.073
LysoPC 18:1	12.5	$[M+H]^+$	522.356	184.073
LysoPC 18:3	12.3	$[M+H]^+$	518.325	184.073
LysoPC 20:0	15.3	$[M+H]^+$	552.403	184.073
LysoPC 20:4	11.7	$[M+H]^+$	544.340	184.073
LysoPC 22:0	16.3	$[M+H]^+$	580.434	184.073

Table S9. List of identified lipidomes in LysoPEs

Identity	Retention time (min)	Adduct ion	Precursor ion (m/z)	Fragment ion (m/z)
LysoPE 16:0	12.6	$[M+H]^+$	454.293	313.293
LysoPE 18:0	14.3	$[M+H]^+$	482.325	341.325
LysoPE 18:1	12.8	$[M+H]^+$	480.309	339.309

Table S10. List of identified lipidomes in TGs

Identity	Retention time (min)	Adduct ion	Precursor ion (m/z)	Fragment ion (m/z)	Fatty acyl chain
TG 42:0	32.6	$[M+NH_4]^+$	740.676	523	C12:0
			740.676	495	C14:0
			740.676	467	C16:0
			740.676	439	C18:0
TG 44:0	33.4	$[M+NH_4]^+$	768.708	467	C18:0
			768.708	495	C16:0
			768.708	523	C14:0
			768.708	551	C12:0
TG 44:1	32.6	$[M+NH_4]^+$	766.692	495	C16:1
			766.692	521	C14:0
TG 46:0	34.1	$[M+NH_4]^+$	796.739	523	C16:0
			796.739	551	C14:0
TG 46:1	33.4	$[M+NH_4]^+$	794.723	495	C18:1
			794.723	523	C16:1
			794.723	549	C14:0
TG 46:2	32.7	$[M+NH_4]^+$	792.708	521	C16:1
			792.708	547	C14:0
TG 48:0	34.8	$[M+NH_4]^+$	824.770	551	C16:0
TG 48:3	32.7	$[M+NH_4]^+$	818.723	547	C16:1
			818.723	521	C18:2
			818.723	573	C14:0
TG 50:0	35.5	$[M+NH_4]^+$	852.802	551	C18:0
			852.802	579	C16:0
			852.802	607	C14:0
TG 50:1	34.6	$[M+NH_4]^+$	850.786	577	C16:0
			850.786	551	C18:1
			850.786	605	C14:0
TG 50:2	34.0	$[M+NH_4]^+$	848.770	549	C18:1
			848.770	603	C14:0
			848.770	577	C16:1
TG 50:3	33.3	$[M+NH_4]^+$	846.755	547	C18:1
			846.755	575	C16:1
TG 50:5	31.6	$[M+NH_4]^+$	842.723	551	C18:5
			842.723	569	C16:0
TG 52:0	36.3	$[M+NH_4]^+$	880.833	607	C16:0
			880.833	579	C18:0
TG 52:1	35.3	$[M+NH_4]^+$	878.817	579	C18:1
			878.817	605	C16:0
			878.817	633	C14:0
			878.817	549	C20:0
TG 52:2	34.5	$[M+NH_4]^+$	876.802	603	C16:0
			876.802	577	C18:1
TG 52:3	33.8	$[M+NH_4]^+$	874.786	603	C16:1
			874.786	601	C16:0
			874.786	551	C20:3
TG 52:5	32.7	$[M+NH_4]^+$	870.755	551	C20:5
			870.755	597	C16:0
TG 52:6	31.7	$[M+NH_4]^+$	868.739	597	C16:1
			868.739	575	C18:4

TG 54:0	37.1	$[M+NH_4]^+$	908.864	607	C18:0
TG 54:1	36.0	$[M+NH_4]^+$	906.848	607	C18:1
			906.848	689	C12:0
			906.848	577	C20:0
			906.848	521	C24:0
			906.848	633	C16:0
			906.848	661	C14:0
TG 54:2	35.1	$[M+NH_4]^+$	904.833	605	C18:1
			904.833	631	C16:0
			904.833	577	C20:1
TG 54:3	34.4	$[M+NH_4]^+$	902.817	603	C18:1
TG 54:4	33.7	$[M+NH_4]^+$	900.802	601	C18:1
			900.802	627	C16:0
			900.802	577	C20:3
TG 54:5	32.9	$[M+NH_4]^+$	898.786	579	C20:5
			898.786	625	C16:0
TG 54:6	32.3	$[M+NH_4]^{++}$	896.770	579	C20:6
			896.770	601	C18:3
			896.770	627	C16:2
			896.770	599	C18:2
			896.770	623	C16:0
			896.770	597	C18:1
			896.770	569	C20:1
			896.770	651	C14:0
			896.770	577	C20:5
TG 56:0	37.7	$[M+NH_4]^+$	936.895	663	C16:0
			936.895	551	C24:0
TG 56:1	36.8	$[M+NH_4]^+$	934.880	635	C18:1
			934.880	605	C20:0
			934.880	689	C14:0
			934.880	661	C16:0
			934.880	577	C22:0
			934.880	549	C24:0
TG 56:6	32.4	$[M+NH_4]^+$	924.802	603	C20:4
			924.802	625	C18:1
TG 58:0	38.8	$[M+NH_4]^+$	964.927	691	C16:0
TG 58:6	33.6	$[M+NH_4]^+$	952.833	603	C22:9
			952.833	607	C22:9
			952.833	653	C18:1
TG 60:0	40.3	$[M+NH_4]^+$	992.958	607	C24:0
			992.958	691	C18:0

Table S11. List of identified lipidomes in DGs

Identity	Retention time (min)	Adduct ion	Precursor ion (m/z)	Fragment ion (m/z)	Fatty acyl chain
DG 30:0	23.2	$[M+NH_4]^+$	558.509	313	C14:0
			558.509	285	C16:0
DG 32:0	25.6	$[M+NH_4]^+$	586.541	313	C16:0
DG 34:0	28.2	$[M+NH_4]^+$	614.572	313	C18:0
			614.572	341	C16:0
DG 34:1	25.3	$[M+NH_4]^+$	612.556	339	C16:0
			612.556	313	C18:1
DG 36:0	30.5	$[M+NH_4]^+$	642.603	341	C18:0
DG 36:5	26.9	$[M+NH_4]^+$	632.525	359	C16:0
			632.525	313	C20:5
DG 38:3	26.9	$[M+NH_4]^+$	664.588	341	C20:3
			664.588	363	C18:0
DG 40:10	18.1	$[M+NH_4]^+$	679.509	313	C24:10
			682.509	443	C14:3
			682.509	409	C16:0
			682.509	283	C26:7
DG 34:5	24.2	$[M+NH_4]^+$	604.494	335	C16:2
			605.494	361	C14:1
			608.494	313	C18:3
			607.494	285	C20:4

Supplementary Figures

Figure S1. Spearman rank correlation of lipids in CVF which were used in our study between PTB and TB

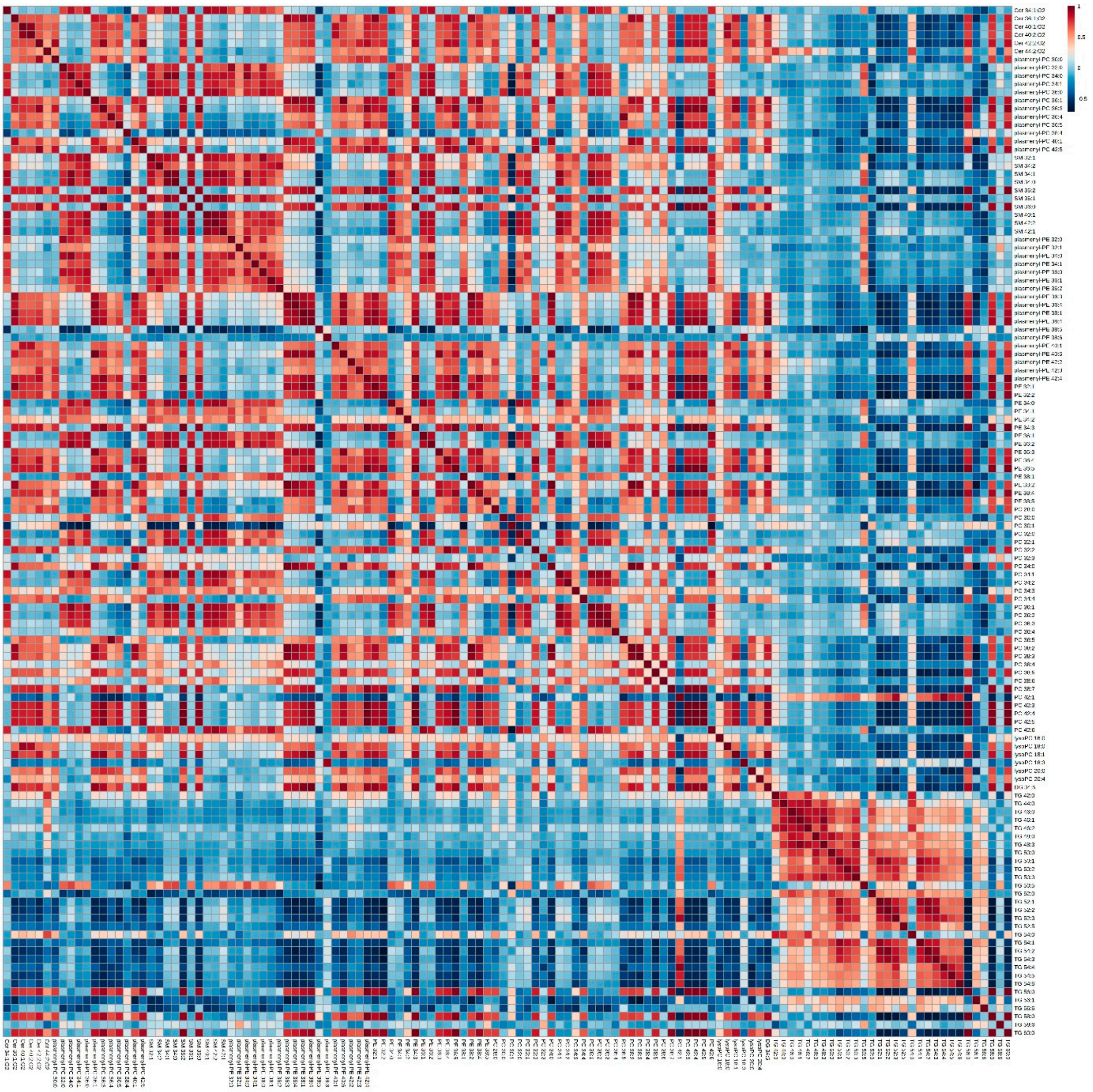

Figure S2. Heats maps representing (A) PlsPCs and PlsPEs, (B) PEs, (C) Cers, (D) SMs, (E) PCs between PTB and TB.

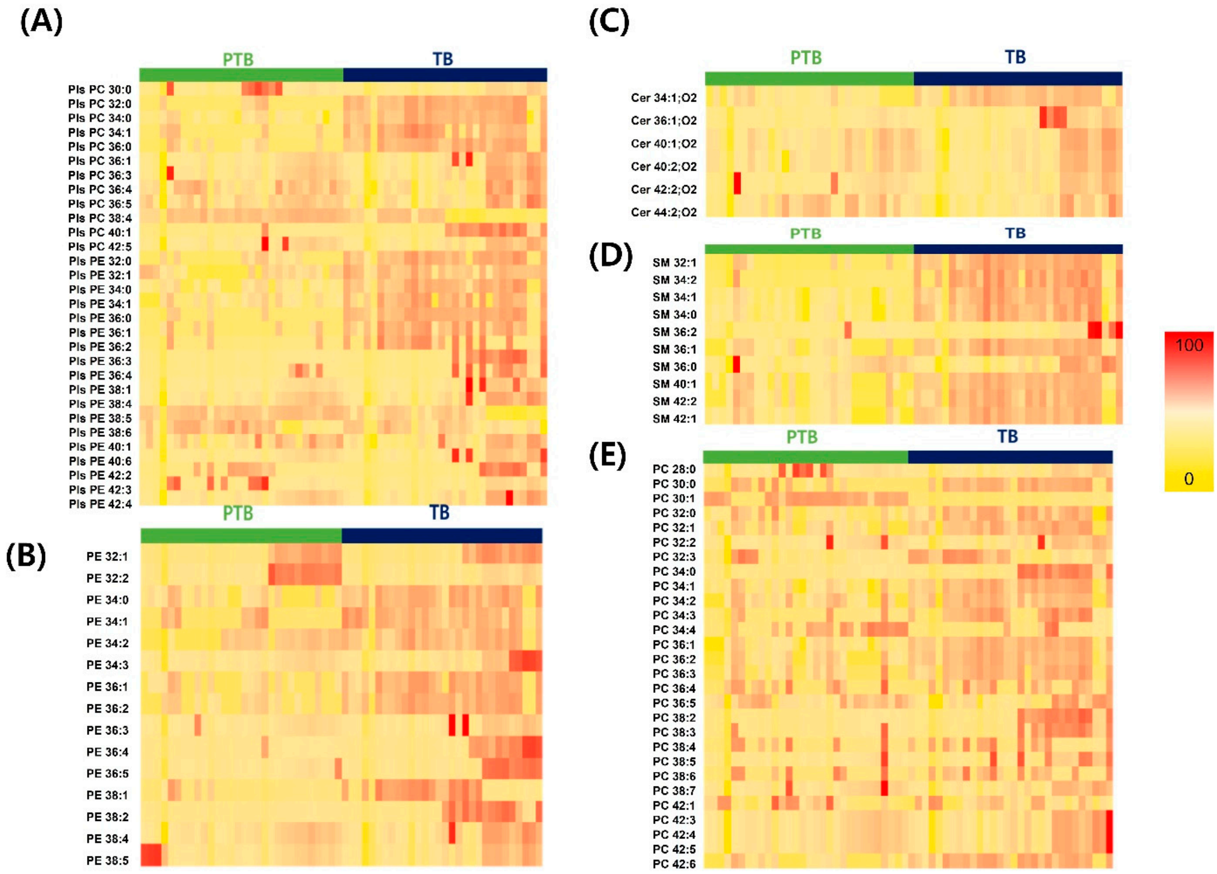

Figure S3. Log 10 peak area of lipidomes in PCs. Orange color in bar graphs and asterisk indicates PTB groups and green color indicates TB. Bar graphs are mean with *p<0.05, **p<0.01, ***p<0.001, and ****0.0001. These are same in Figure S3-S10

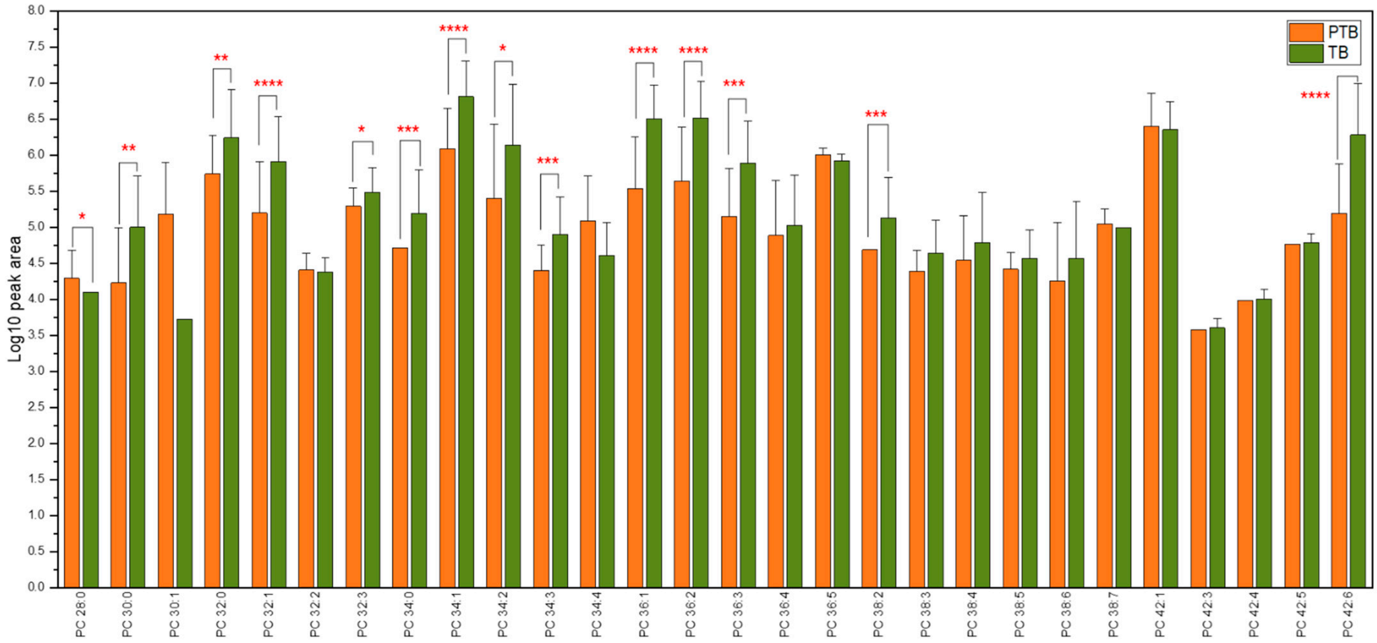

Figure S4. Log 10 peak area of lipidomes in PEs

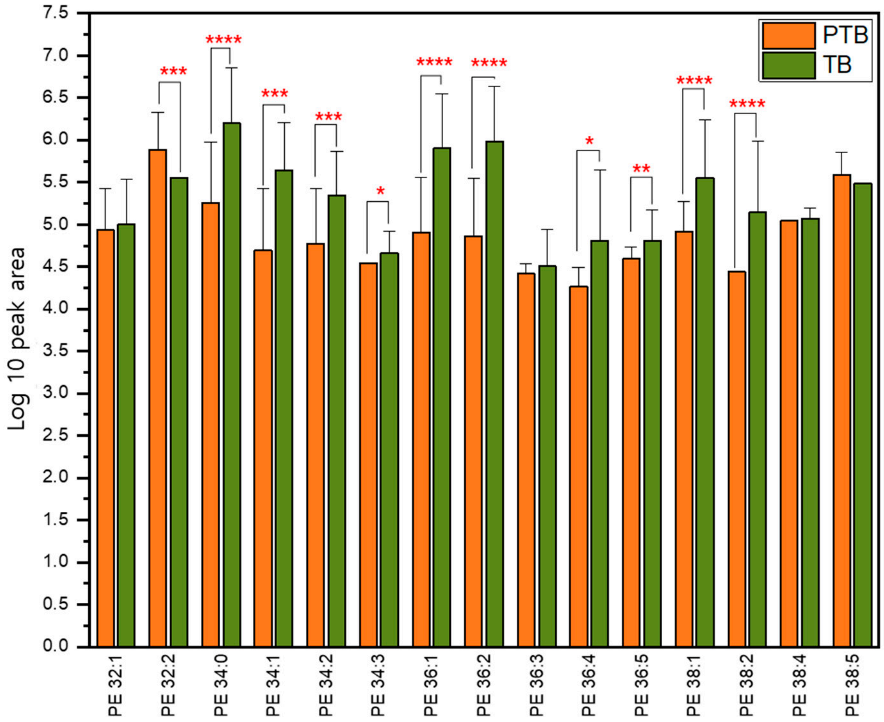

Figure S5. Log 10 peak area of lipidomes in PlsPCs

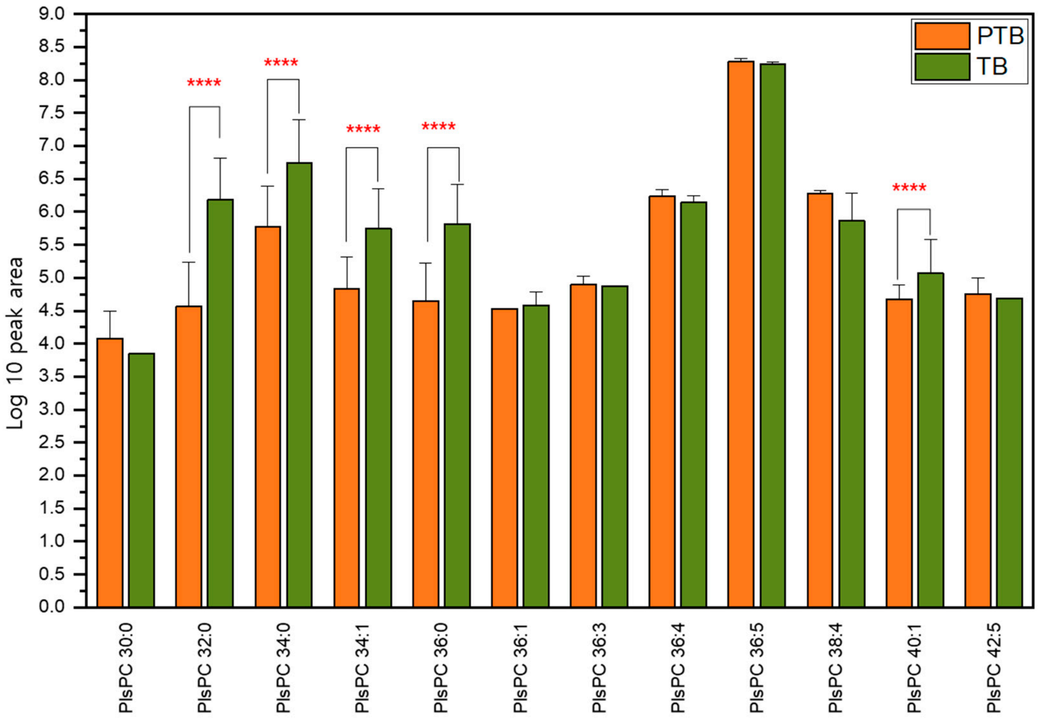

Figure S6. Log 10 peak area of lipidomes in PlsPEs

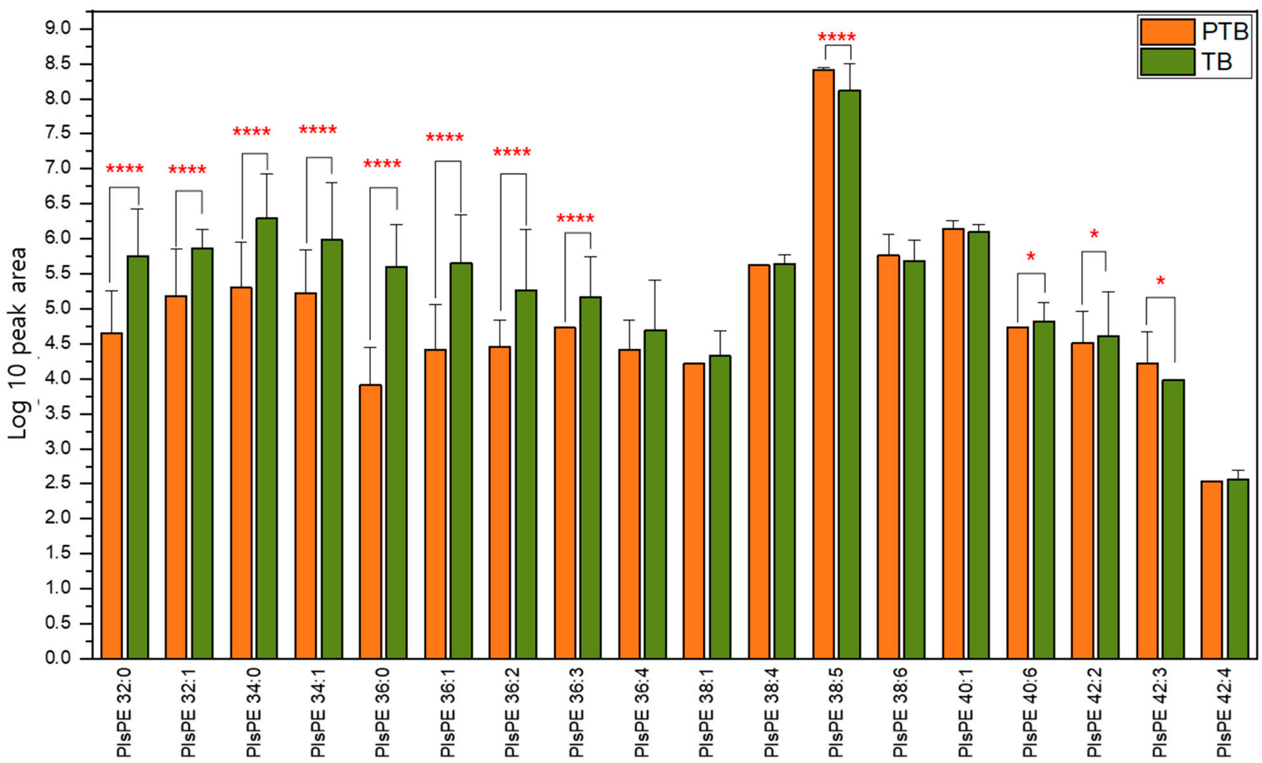

Figure S7. Log 10 peak area of lipidomes in SMs

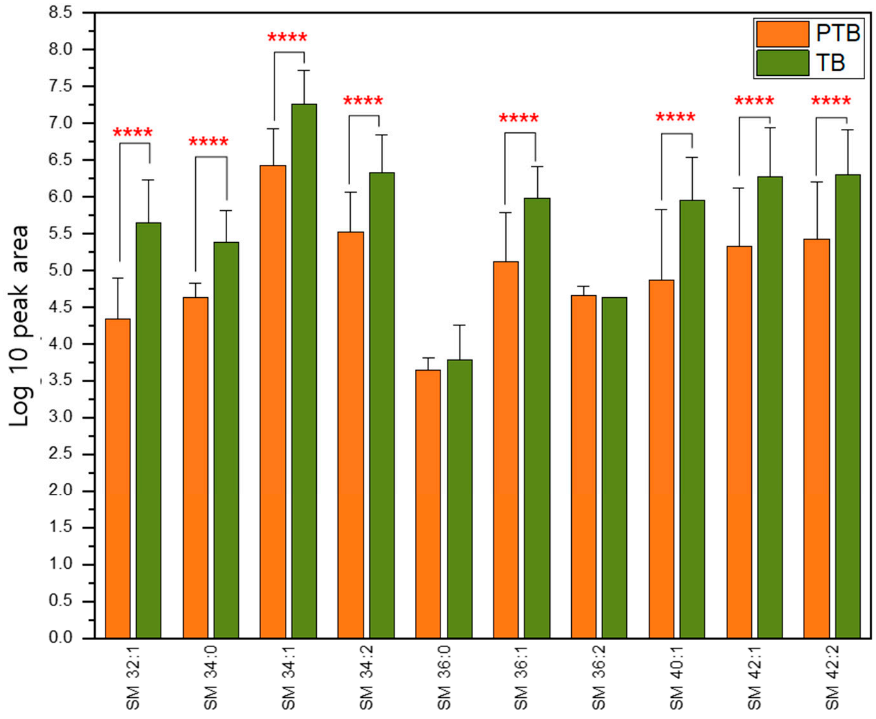

Figure S8. Log 10 peak area of lipidomes in Cers

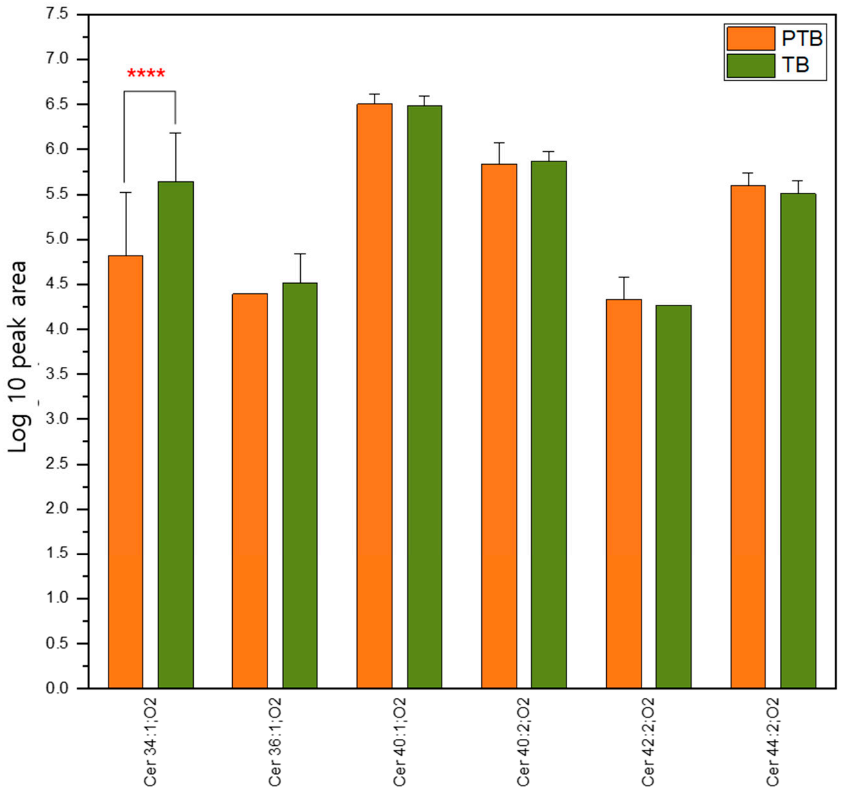

Figure S9. Log 10 peak area of lipidomes in TGs

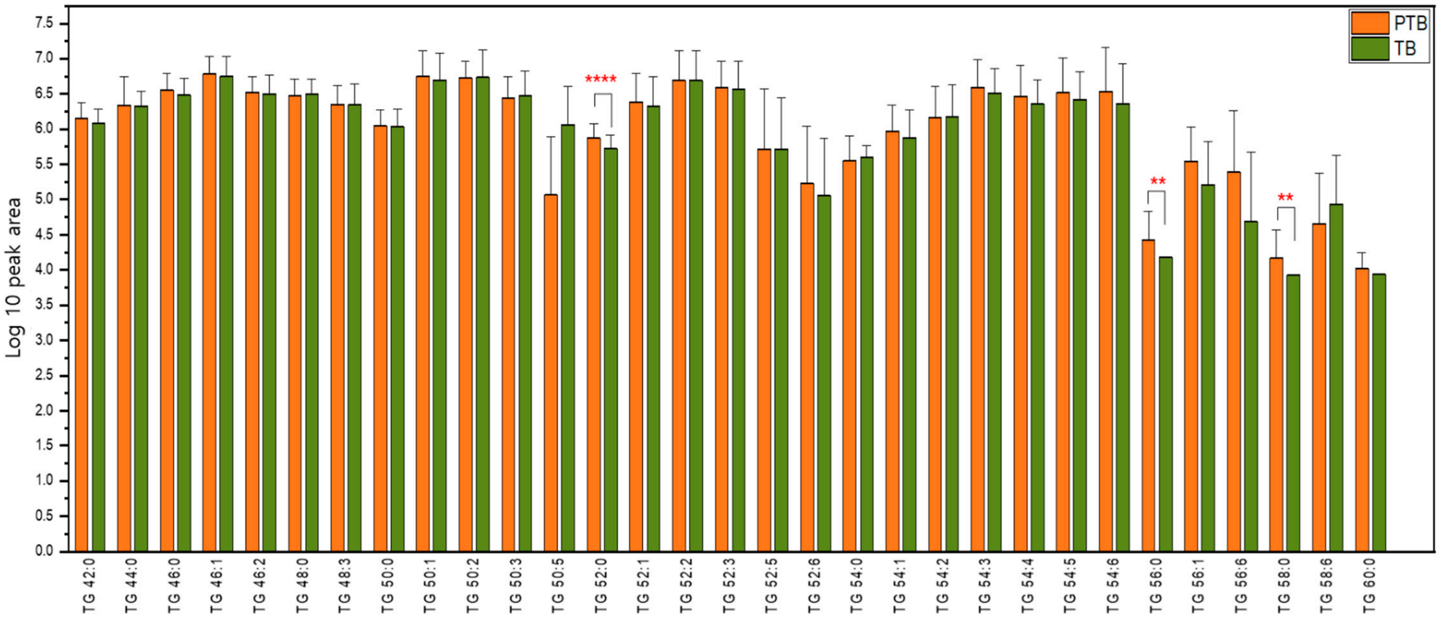

Figure S10. Log 10 peak area of lipidomes in LPCs

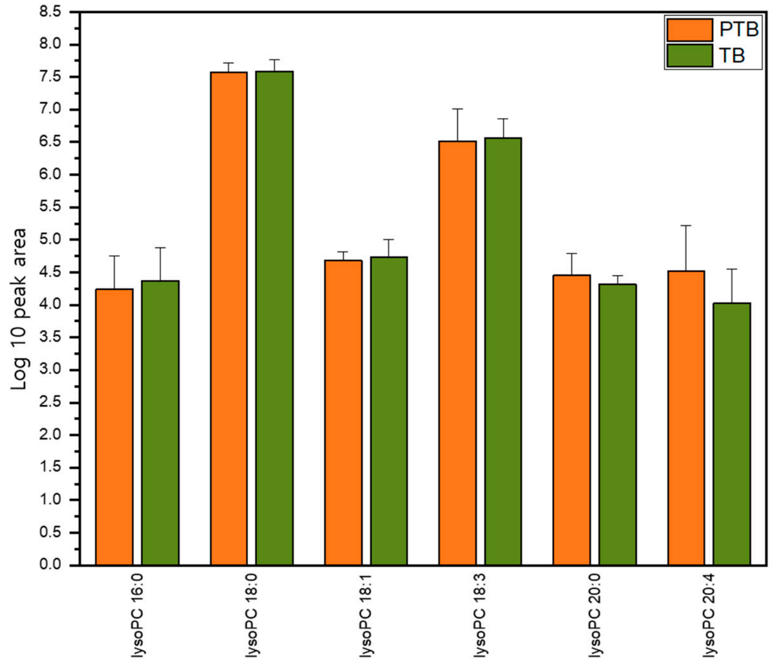

Supplement: Supplementary file 1 [file metabolites-13-00177-s001.zip › Supplementary information_221219.pdf]
